# Supplementary material for: Membrane shape-mediated wave propagation of cortical protein dynamics
Source: Nat Commun. 2018 Jan 10;9:136. doi: 10.1038/s41467-017-02469-1 (PMC5762918; doi:10.1038/s41467-017-02469-1)
Supplement: Supplementary file 1 — Supplementary Information [file 41467_2017_2469_MOESM1_ESM.pdf]

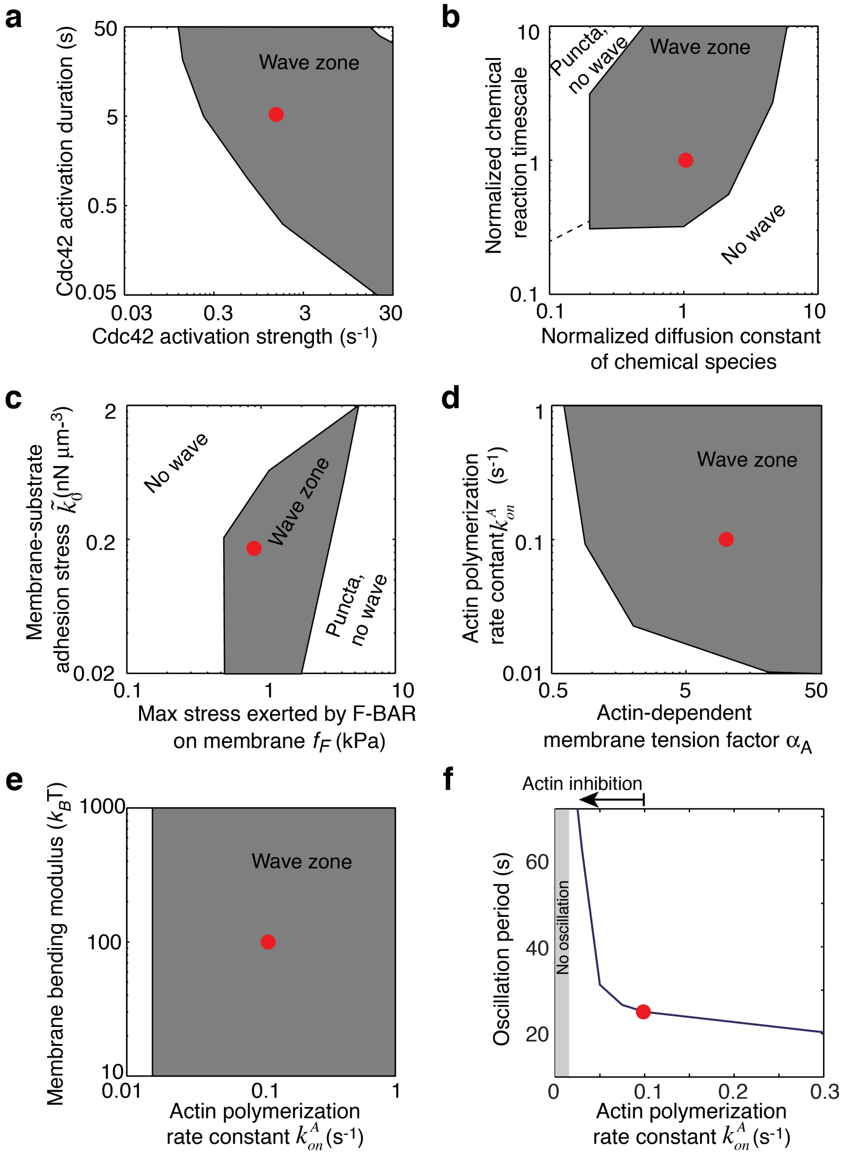

Supplementary Figure 1. Model phase diagrams showing that traveling waves are preserved over a broad range of model parameters: (**a**) Cdc42 activation duration and strength; (**b**) chemical reaction timescale and diffusion constant of chemical species: the time scales and diffusion constants were changed for all chemical species at the same rate and normalized by nominal values; (**c**) the stress exerted by F-BAR and the membrane-substrate adhesion; (**d**) actin polymerization-dependent membrane tension and actin polymerization rate; (**e**) membrane bending modulus and actin polymerization rate. (**f**) Oscillation periods are prolonged upon the inhibition of actin polymerization. The red dot in each diagram (a-f) represents the nominal model parameter value.

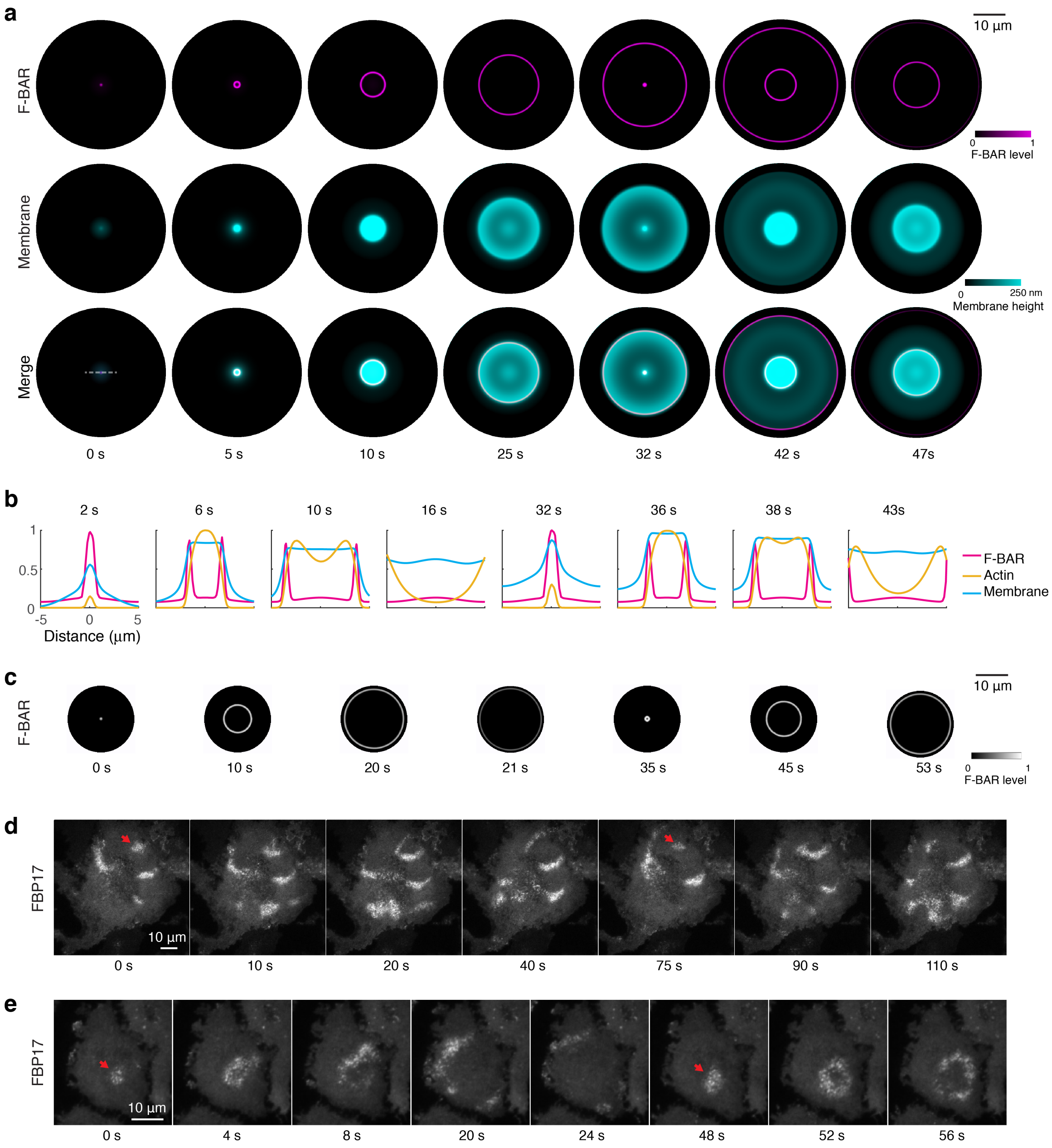

Supplementary Figure 2. Snapshots of traveling waves. (**a**) Snapshots of predicted wave trains of cortical F-BAR and membrane height across the simulation domain. *Upper*: F-BAR level (density map in magenta). The color bar scales the local F-BAR concentration normalized to its maximum. *Middle*: Membrane height (density map in cyan). The color bar scales the local membrane height (in nm). *Lower*: Merged density maps of F-BAR level (magenta) and membrane height (cyan). Overlap color displayed in white. (**b**) Zoom-in profiles of cortical F-BAR, actin, and membrane shape along the cross-section near the epicenter (indicated by gray dashed line in (a)). These snapshots are derived from the same simulation results in (**a**). F-BAR level, actin level, and membrane height are normalized to their respective maximum values. Importantly, after the first F-BAR wave has passed (from t = 6 s to 16 s), the actin level returns to baseline at the epicenter.

Because actin level modulates cortex tension, cortex tension is much lower at the epicenter than in the periphery. Accordingly, membrane shape relaxes more slowly, resulting in a "residual" membrane bump at t = 16 s, even though most of the protein wave has passed. This residual membrane shape deformation both promotes, and is further promoted by, F-BAR cortical recruitment from t = 16 s to 32 s, and beyond, giving rise to the oscillatory dynamics (*i.e.*, the profiles at t = 36 s repeat that at t = 6 s). (**c**) Snapshots of predicted single-round of cortical F-BAR profile and membrane shape across a smaller simulation domain. Here, we used the same model parameters as in (**a**) except the simulation domain was smaller (20 μm in diameter instead of 40 μm). Due to the smaller domain size the first wave disappears at the boundary before a second wave initiates at the epicenter; a single wave propagates, rather than wave trains. (**d**) and (**e**) Experimental observations of wave trains and single waves, similar to those predicted in (**a**) and (**c**), respectively. The red arrows in (**d**) and (**e**) point to newly initiating waves. Scale bars: 10 μm.

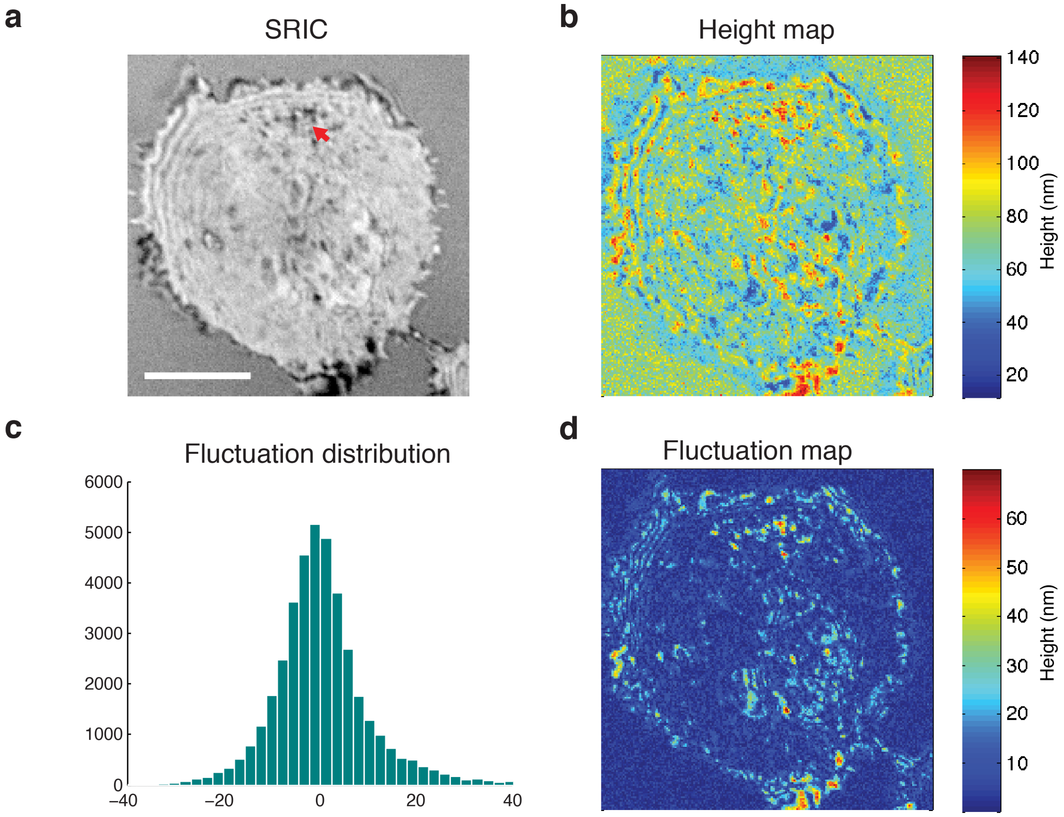

Supplementary Figure 3. Membrane height estimation from SRIC measurement. (**a**) SRIC wave shown by red arrow. (**b**) Height map. We inferred the membrane height from the equation $h = \frac{\lambda}{4\pi n_1}\left(\sin^{-1}\left(\left(2I - I_{\max} - I_{\min}\right)/\left(I_{\max} - I_{\min}\right)\right) + \frac{\pi}{2}\right)$ as modified from reference [1]. This is similar to Fig. 5a, *middle* in the paper [1]. (**c**) Fluctuation distribution, and (**d**) Fluctuation map, where fluctuations are defined by the equation: $u = \frac{I(t) - <I>}{\sqrt{<I>}}$. Scale bar: 10 μm.

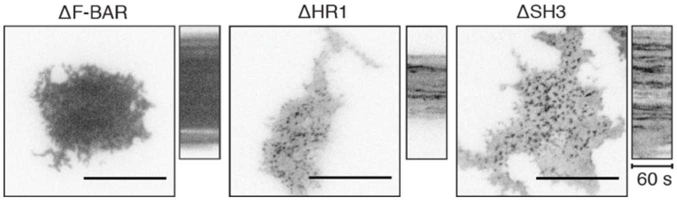

Supplementary Figure 4. Domain-deleted mutants of FBP17 do not generate waves. Δ HR1 and Δ SH3 mutants formed puncta with long lifetimes, while fluorescence from the Δ F-BAR mutant remained diffuse. Scale bar: 10 μm.

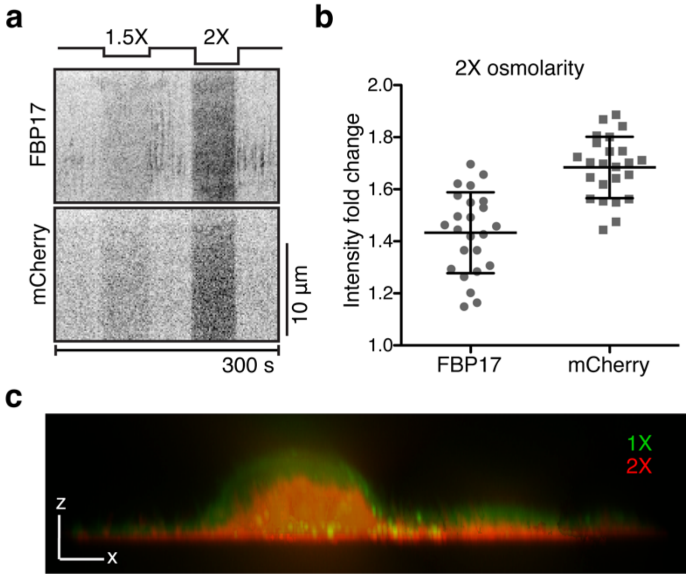

Supplementary Figure 5. Increased FBP17 intensity upon hyper-osmotic shock is due to volume effect. (**a**) Kymograph of FBP17-GFP and soluble mCherry co-expressed in the same cell subjected to osmotic shock. (**b**) Scatter plot of FBP17-GFP and soluble mCherry TIRF intensity in 2X osmolar–buffer compared to isosmotic (1X) buffer in single cells (n=24 cells). (**c**) XZ view of confocal Z-stack of an RBL cell expressing soluble GFP shows the cell volume is reduced about 50% in 2X buffer, while there is little change in cortical surface area. Scale bar: 10 µm.

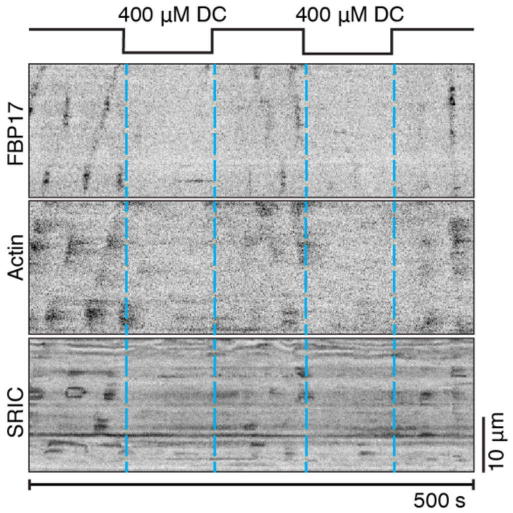

Supplementary Figure 6. Desoxycholate (DC) shock inhibits FBP17, actin, and membrane height waves. Kymographs of FBP17-GFP, mCherry-actin, and SRIC images of the same cell cyclically subjected to 400 µM DC. The schematics above the kymographs shows when DC was present and putative changes in cell membrane tension.

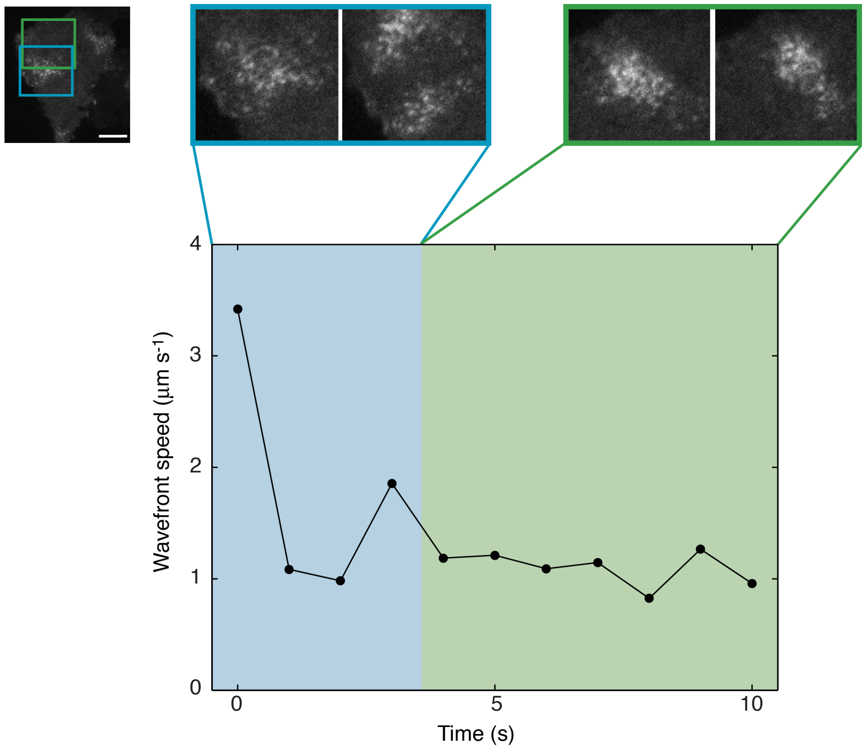

Supplementary Figure 7. Evolution of instantaneous wave speed. The time curve (black line) shows a typical temporal evolution in the wave speed of F-BAR traveling waves. The blue color indicates the nucleation stage, and the green color indicates the wave propagation stage. The upper left corner shows the snapshot of the whole cell at $t = 0$ s, in which the blue box and the green box mark the location of the zoom-in snapshots shown above the time curve. Scale bar: 10 μm.

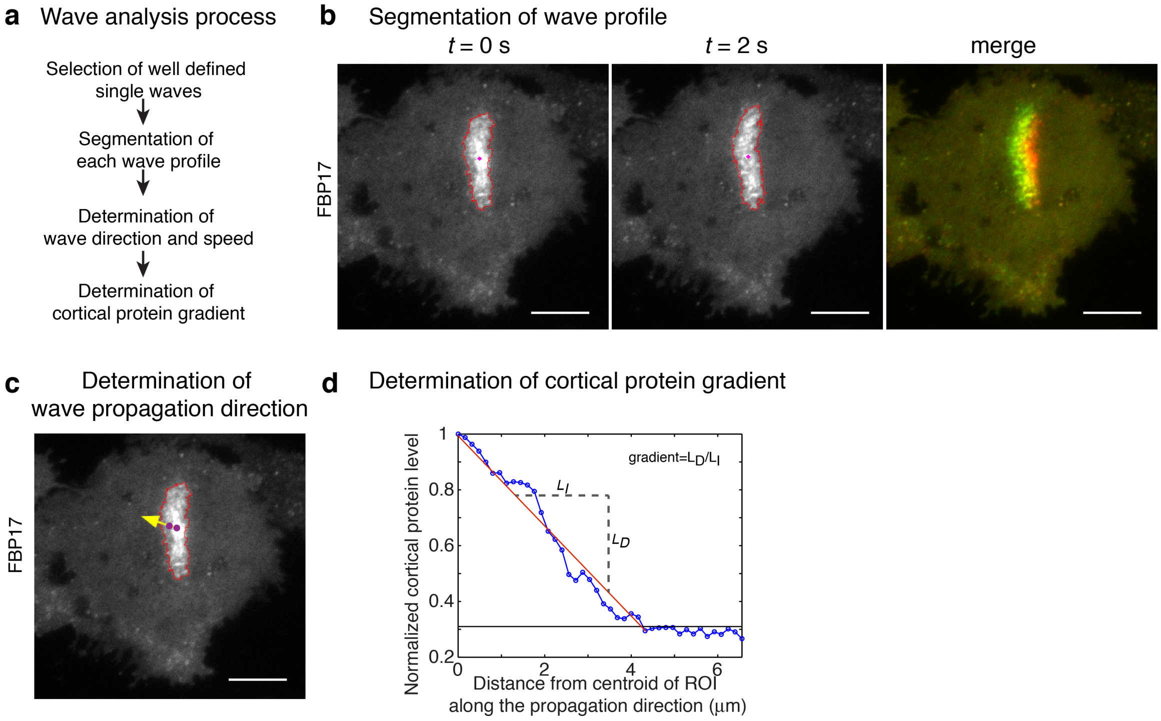

Supplementary Figure 8. Procedure for measurement of wave speed and cortical protein gradient. (**a**) The wave analysis process. (**b**) Segmentation of wave profile. TIRF images of florescence of FBP17. Typical wave snapshots with wave outline marked. *Left*: the wave profile at $t = 0$ s, *middle*: $t = 2$ s, *right*: merged intensity map of $t = 0$ s image in red and 2 s image in green. The purple dots in the left and middle panel indicate the centroids of the wave at each time frame. (**c**) Determination of wave speed and propagation direction. The wave speed is determined by measuring the distance between centroids (filled purple circles) in neighboring frames along time. The arrow indicates the direction of the wave propagation. (**d**) Determination of cortical protein gradient. The intensity profile along the direction of the wave propagation indicated in (c) from the centroid of the wave at time $t = 0$ s. The red line indicates the linear fit from the centroid to the background intensity. The gradient of normalized protein level at wavefront is determined by calculating the slope of the red line L_D/L_I. The intensity is normalized to the maximum intensity of the measured cell. Scale bar: 10μm.

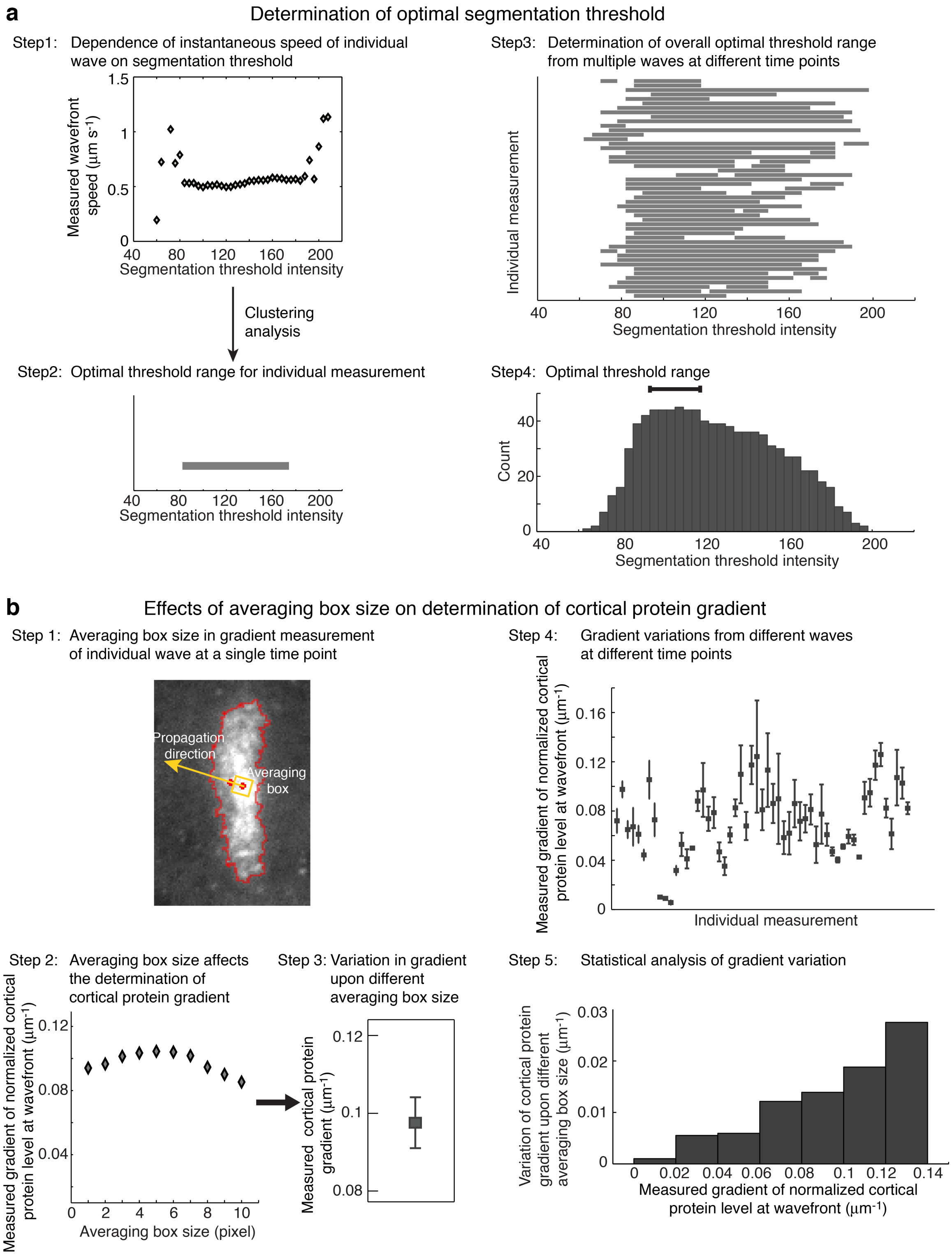

Supplementary Figure 9. Robustness of tracking results. (**a**) Determination of optimal segmentation threshold. Step 1: Dependence of instantaneous speed of individual wave on segmentation thresholds. Step 2: Clustering analysis determines the optimal threshold range for each individual measurement. The line indicates the two longest clustered regions, where the neighboring measured speed variation less than 0.1 μm s^{-1} within

single region. Step 3: Determination of overall optimal threshold range from multiple waves at different time points. The plot shows the measurement of 2 waves. Step 4: The distribution of optimal region for all measurements in step 3. The region with the highest occurrences is chosen to be the overall optimal range. (**b**) The effects of the averaging box size on determination of the cortical protein gradient at wavefront. Step 1: Averaging box size in gradient measurement of individual wave at a single time point. The red line indicates the outline of the analyzed wave. The red dots indicate the two centroids at the sequential time points. Yellow arrow indicates the direction of wave propagation. Yellow square marks the averaging box. Step 2: Averaging box size affects the determination of cortical protein gradient. Step 3: Variation in gradient upon different averaging box sizes for an individual wave at a single time point. Step 4: Gradient variation from different waves at different time points. Step 5: Uncertainty in the measured cortical protein gradient as a function of gradient magnitude. Error bars: s.d.

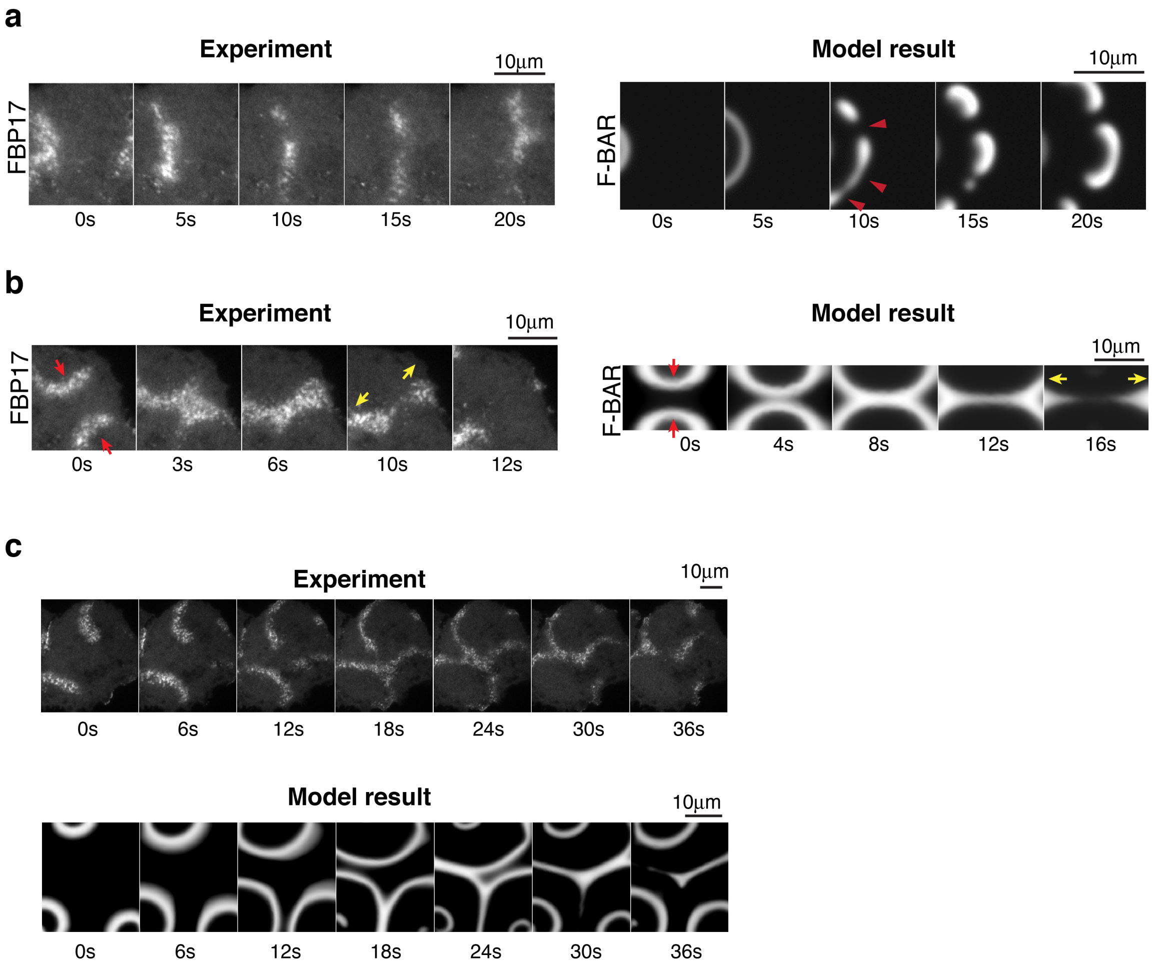

Supplementary Figure 10. Representatives of complex wave patterns observed in experiments and the corresponding model results. (**a**) Coherent propagating wave splits into segments. In the right panel, transient perturbations that inhibited the local membrane shape deformation were introduced in the model (indicated by red arrows) between time 5 s to 15 s. These perturbations mimicked effects of stochastic fluctuations. All other parameters and conditions were the same as in the nominal case of Fig. 1. (**b**) Two waves collide and then propagate in the directions orthogonal to their incoming paths. The red arrows indicate the wave propagation direction before colliding and the yellow arrows indicate the direction after colliding. (**c**) Three-wave collision.

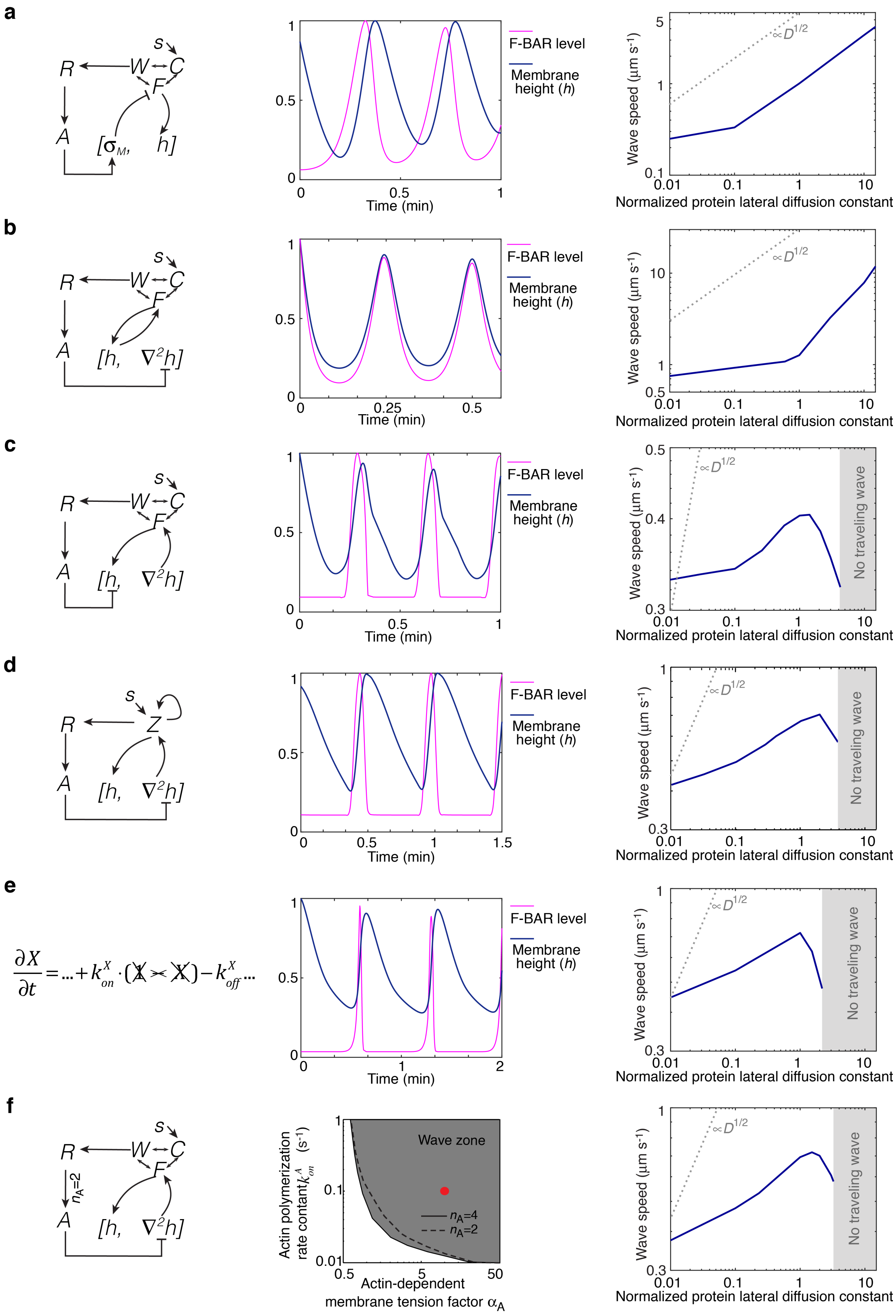
a
b
c
d
e
f
F-BAR level
Membrane height (h)
Time (min)
Wave speed (μm s-1)
Normalized protein lateral diffusion constant
∝D1/2
No traveling wave
Wave zone
nA=4
nA=2
nA=2
Actin polymerization rate contant kAon (s-1)
Actin-dependent membrane tension factor αA

Supplementary Figure 11. Alternative model schemes with curvature-sensing effects preserve the essential conclusions from our nominal model. (**a**) Alternative mechanochemical model with membrane tension-modulated F-BAR dynamics. This altered model scheme is without curvature-sensing effect. Instead, the F-BAR cortical recruitment is only promoted by the CWF module, and membrane tension speeds up the F-BAR turnover from the cortex. (**b**) Alternative mechanochemical model with membrane height-mediated F-BAR cortical recruitment. The altered model scheme, again, is without curvature-sensing effect. The F-BAR cortical recruitment was promoted by h, rather than $-\nabla^2 h$ (Eq. (3.3)). (**c**) Mechanochemical model with actin polymerization directly strengthening membrane-substrate adhesion. Here, the curvature-sensing effect is preserved as in our nominal model scheme. (**d**) Nominal model scheme that simplifies CWF module by one component, *Z*. (**e**) Nominal model scheme without the terms (1-*X*). Here, we replaced term (1-*X*) in Eqs. (1.1-1.5) by just 1, where *X* represents the variables in the model (*C, W, F, R, A*) (See Eqs. (3.5-3.9)). (**f**) Nominal model scheme with a lower nonlinearity for actin dynamics. n_A=2 instead of 4 as in the nominal case. For (**a**-**e**): *Left*: Schematics of altered model schemes. *Middle*: Temporal evolutions of local F-BAR cortical level (magenta line) and membrane height (blue line) at the fixed location along the wave propagation path. Both F-BAR level and membrane height were normalized to their respective maximum values. *Right*: Diffusion-dependence of wave speed. For (**f**): *Left*: Schematics of the altered model scheme. *Middle*: Model phase diagram shows the wave zone changes when the Hill coefficient, n_A, in the equation of Arp2/3 complex-mediated F-actin polymerization changes from 4 (solid line) to 2 (dashed line). *Right*: Diffusion-dependence of wave speed. In all the model variations, the parameters are the same as in the nominal case in the main text except for those listed in Supplementary Table 3. For the all individual results in (**a**-**f**), their essential features are representative of their corresponding models that persist in a broad parameter space.

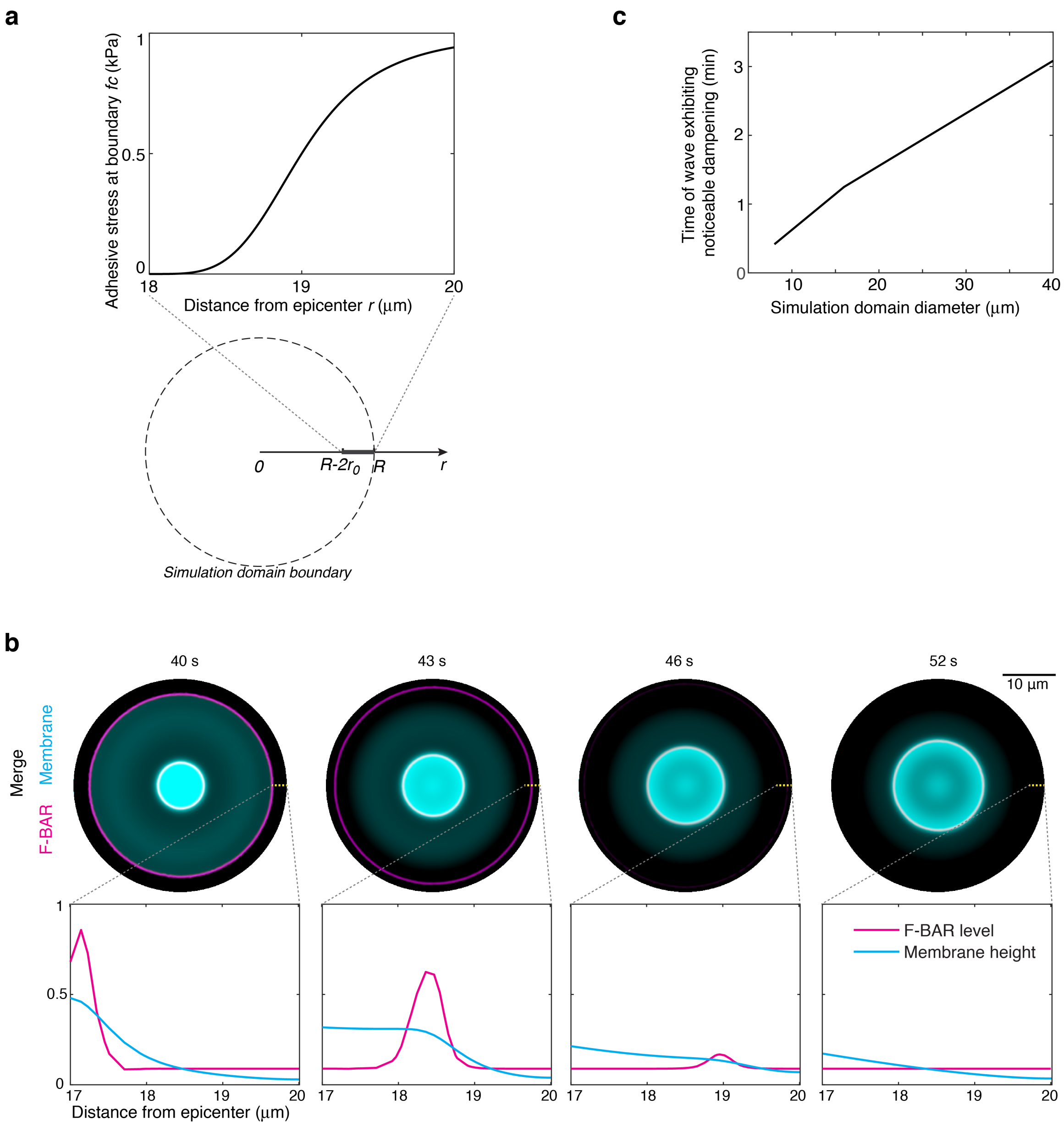

Supplementary Figure 12. Clamping the membrane at the boundary in the model dampens the wave. (**a**) Clamped boundary condition is implemented by a spatially-dependent adhesive stress (see our elaboration on the boundary condition in the Supplementary Note 1). (**b**) Clamped boundary condition disrupts traveling wave. *Upper*: Merged snapshots of F-BAR level (magenta) and membrane height (cyan) across the entire simulation domain when the wave approaches the boundary. *Lower*: Zoom-in profiles of F-BAR level and membrane height along the cross-section near boundary (indicated by the yellow dashed line in upper panel). F-BAR level and membrane height are normalized to their respective maximums. (**c**) Clamping the membrane at the boundary in our model dampens oscillation dynamics over time. The y-axis represents the time when the oscillation magnitude of the membrane height has decreased by 30% as

compared to the maximum value at the epicenter. In all cases, the traveling waves eventually will disappear, consistent with our experimental observations that the individual traveling waves are not strictly sustained oscillation and disappear in long times (*e.g.*, see Supplementary Fig. 13). Here, our model results show that the larger the simulation zone, the longer it takes for the effect of clamped boundary to dampen the oscillation amplitude. All the experimental data points in this paper were taken long before the waves approached the cell edge.

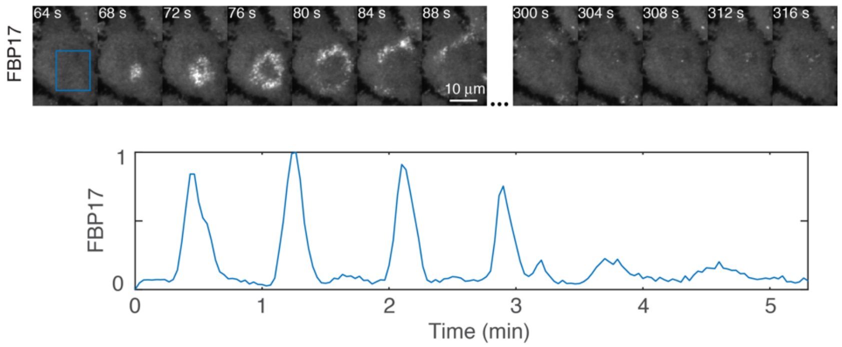

Supplementary Figure 13. A representative experimental observation showing the dampening of oscillations over time in the absence of interference by other waves. *Upper*: Snapshots of a single wave propagation. The density map shows the FBP17 level. *Lower*: Temporal evolutions of the local FBP17 density as marked by the blue box in the upper panel. The FBP17 level in lower panel is normalized to its maximum value after subtracting the background. Scale bar: 10 μm.

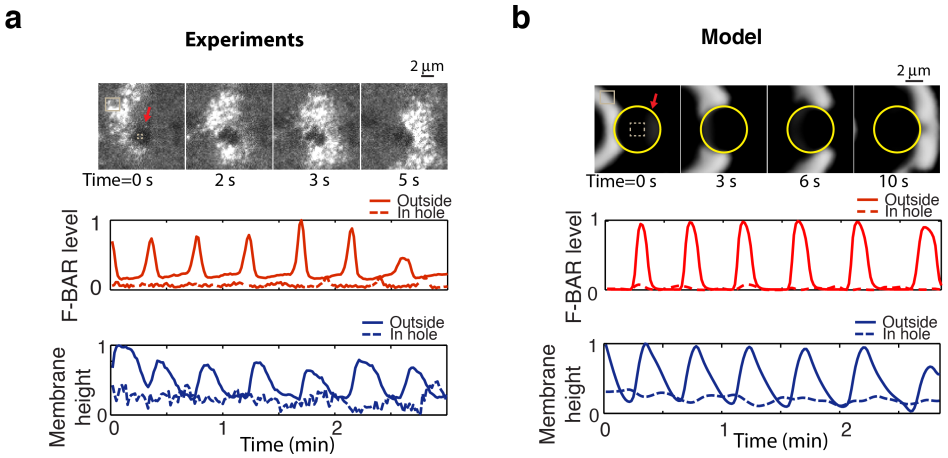

Supplementary Figure 14. Traveling waves circumvent the cortical area with strong membrane-substrate adhesion that clamps the membrane. (**a**) Experiment observations. The local F-BAR level is measured by TIRFM and the relative membrane height is inferred from SRIC experiments. (**b**) Model results from simulation that mimicked the strong local membrane-substrate adhesion effect. *Upper*: Snapshots of FBP17-GFP wave. *Middle* and *lower*: Time curves of the local F-BAR levels and relative membrane height at the fixed locations inside and outside the "hole" marked by the grey squares, respectively. Here, the hole is the area where local membrane does not undergo notable shape changes, marked by the red arrow (experiment), and the red arrow and yellow circles (model).

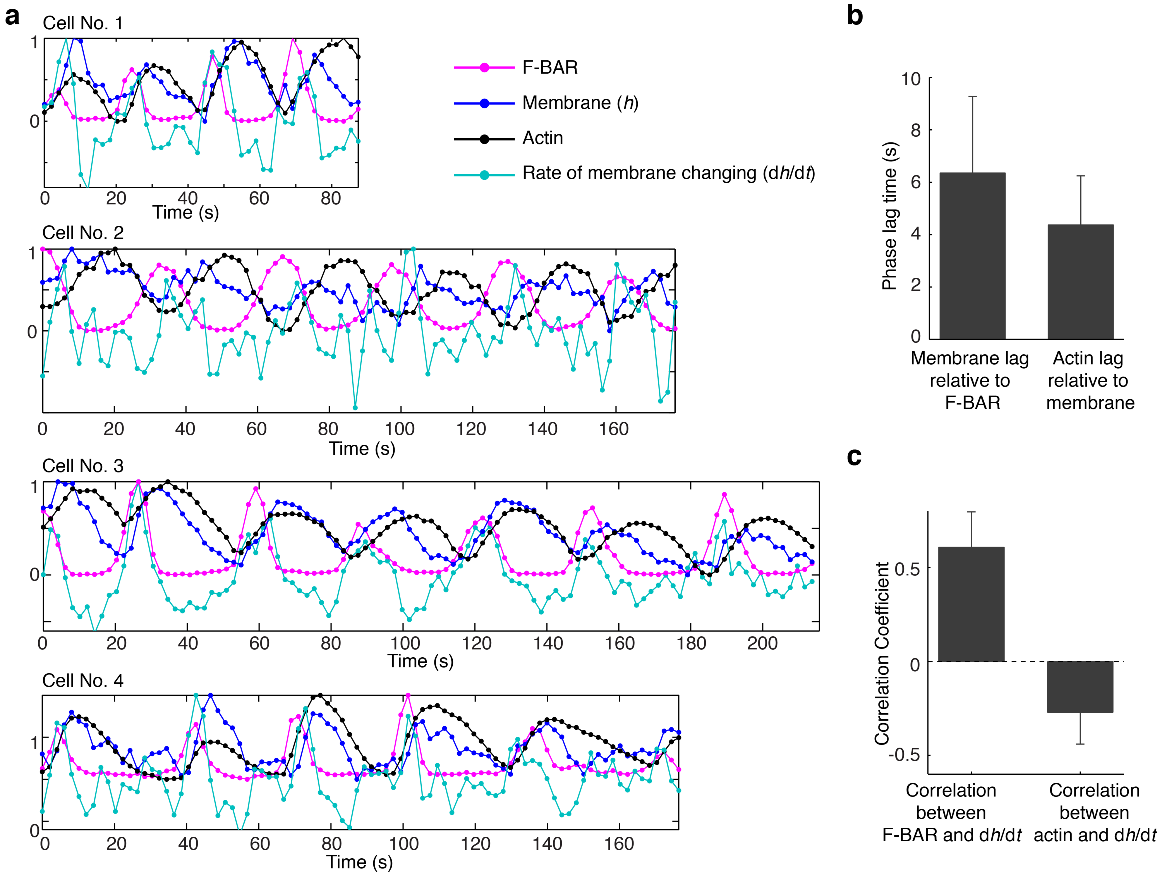

Supplementary Figure 15. F-BAR drives membrane deformation instead of actin. (**a**) Measurements of local oscillations demonstrate the phase lag behavior of F-BAR, membrane (SRIC), actin, and rate of change in membrane height (d*h*/d*t*). The plots show that membrane oscillation has a phase lag relative to F-BAR and actin oscillation has a phase lag relative to membrane. The rate of membrane changing is in phase with F-BAR instead of actin. Each panel shows the measurement from one individual cell. Purple line: normalized F-BAR level; blue line: normalized membrane height; black line: normalized actin level; cyan line: rate of change in membrane height d*h*/d*t*. (**b**) Quantification of the mean phase lag time of measurements in (a). N=4 cells. Error bars: s.d. (**c**) Quantification of correlation coefficients between F-BAR and d*h*/d*t*, and between actin and d*h*/d*t*. N=4 cells. Error bars: s.d.

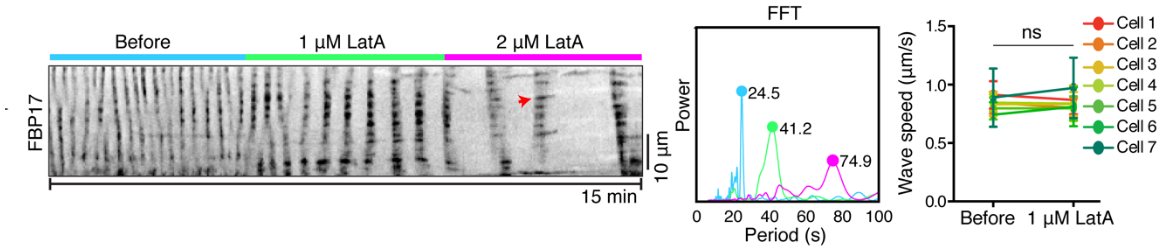

Supplementary Figure 16. Propagation speed of waves is independent of oscillatory periodicity. *Left*, Kymograph of an RBL cell with FBP17-GFP waves treated with titration of Latrunculin A (LatA). Red arrow shows an individual punctum. *Middle*, Fast Fourier transform (FFT) shows the dominant periods (colored dots) of the same cell under each treatment. Colors indicate each treatment as in *Left*. *Right*, Wave speed was not significantly changed after 1 µM LatA treatment. Each pair represents a single cell before and after LatA. Error bar is standard deviation (s.d.) of wave speed within each cell. (n = 4 experiments; ns: no significant difference within each cell; student t-test).

Supplementary Tables

Supplementary Table 1. Parameters in mechanics.

Parameter Symbol	Parameter description	Measured or estimated value	Value used in the model	Reference
κ_m	Membrane bending modulus	4.2×10^{-19} J	4.2×10^{-19} J	2, 3
σ_0	Membrane surface tension	~ 10^{-4} – 10^{-5} J m^{-2}	1×10^{-4} J m^{-2}	2, 3, 4, 5, 6 (Note (*))
λ_m	Membrane viscous drag coefficient	~ 2×10^{9} Pa s m^{-1}	2×10^{9} Pa s m^{-1}	7
f_F	Maximum stress exerted by F-BAR		750 Pa	Estimated
$\tilde{k}_0$	Membrane-substrate adhesion stress	~0.05-0.25 nN µm^{-3}	0.25 nN µm^{-3}	2
Ω_0	Characteristic membrane curvature that recruits F-BAR	-1/50 – -1/200 nm^{-1}	-1/200 nm^{-1}	8, 9, 10

Supplementary Table 2. Parameters in chemical reactions.

Parameter Symbol	Parameter description	Measured or estimated value	Value used in the model	Reference
D_C	Diffusion constant for Cdc42	~ 0.02 – 0.06 µm^2 s^{-1}	0.05 µm^2 s^{-1}	11, 12, 13, 14
D_W	Diffusion constant for WASP family proteins	~ 0.35 – 0.45 µm^2 s^{-1}	0.4 µm^2 s^{-1}	15
D_F	Diffusion constant for F-BAR	~ 0.13 – 0.63 µm^2 s^{-1}	0.3 µm^2 s^{-1}	16, 17
D_R	Diffusion constant for Arp2/3	~ 0.58 – 0.68 µm^2 s^{-1}	0.6 µm^2 s^{-1}	15
k_{on}^{C}	Activation rate constant of Cdc42	~0.05-5 s^{-1}	2.5 s^{-1}	18
k_{off}^{C}	De-activation rate constant of Cdc42	~0.05-5 s^{-1}	3 s^{-1}	18
k_{on}^{W}	Activation rate constant of N-WASP	0.1-3 s^{-1}	2.5 s^{-1}	19
k_{off}^{W}	De-activation rate constant of N-WASP	0.1-3 s^{-1}	3 s^{-1}	19
k_{on}^{F}	Recruitment rate constant of F-BAR		0.3 s^{-1}	Estimated
k_{on}^{Fh}	Curvature-dependent recruitment rate of F-BAR		30 s^{-1}	Estimated
k_{off}^{F}	Turnover rate constant of F-BAR into cytoplasm	~0.5-2 s^{-1}	3 s^{-1}	16
k_{on}^{R}	Recruitment rate constant of Arp2/3	0.1-1 s^{-1}	0.6 s^{-1}	19, 20
W_0	Normalized threshold N-WASP level to promote Arp2/3 activation	0-1	0.7	19, 21
k_{off}^{R}	De-activation rate constant of Arp2/3	0.1-1 s^{-1}	0.2 s^{-1}	19, 20
k_{on}^{A}	Actin polymerization rate	0.05-0.25 s^{-1}	0.1 s^{-1}	22, 23, 24, 25
R_0	Characteristic Arp2/3 activity to promote actin polymerization	0-1	0.25	19, 20, 26, 27
k_{off}^{A}	Actin depolymerization rate	0.05-0.25 s^{-1}	0.1 s^{-1}	22, 23, 24, 25
α_A	Maximum fold increase in actin-mediated membrane tension	2-10	5	28, 29
A_0	Threshold actin level to modulated membrane tension	0-1	0.2	30, 31

Note (*): In the paper by Dai *et al.* [4], they used a tether to pull out the membrane tubule, from which the static tether force (F_0) was measured at equilibrium. The initial pulling force (Fig 1C in ref. [4]) or variations of tether force between individual cells (Fig 4 in ref. [4]) can be at least twice of the average tether force. The static tether force relates to the membrane tension σ and the bending modulus κ by $F_0 = 2\pi(2\sigma\kappa)^{1/2}$. As Dai et al stated in their paper, the membrane tension was estimated to be 0.025 mN m^{-1} at resting state, by assuming the bending modulus $\kappa \sim 2.7 \times 10^{-19}$

N·m, which was measured from the neuronal growth cone [32]. Note that this bending modulus of 2.7×10^{-19} N·m is not from RBL cells. Since the membrane bending modulus of RBL cells was not measured independently in that work, the membrane tension is an estimated value. The measured membrane bending modulus ranges from 10 to 100 $k_B T$ (*i.e.*, 4.2×10^{-20} N·m to 4.2×10^{-19} N·m) [2, 3, 33, 34], depending on the model systems. Based on the range of measured membrane bending modulus and the tether force measured by Dai *et al.* [4], we estimated the RBL cell membrane tension at resting state to be 0.16 to 0.016 mN m^{-1}. The model nominal value for membrane tension 0.1mN m^{-1} is thus within this estimated range.

Supplementary Table 3. Parameters in alternative model schemes.

Parameter Symbol	Parameter description	Measured or estimated value	Value used in the model	Reference
k_{on}^{F*}	Recruitment rate constant of F-BAR in the alternative model 3.1		30 s^{-1}	Estimated
k_{off}^{F*}	Rate constant for release of F-BAR into the cytoplasm in the alternative model 3.1		1.5 s^{-1}	Estimated
λ_A^*	Timescale change for actin dynamics in the alternative model 3.1		0.3	Estimated
h_{00}^*	Characteristic membrane height that recruits F-BAR in the alternative model 3.2		1 µm	Estimated
k_{on}^{Z}	Recruitment rate of Z in the alternative model 3.4		3 s^{-1}	Estimated
Z_1	Normalized threshold Z level to promote Arp2/3 activation in Eq. (2.2)		0.2	Estimated
k_{on}^{Fh*}	Curvature-dependent recruitment rate of F-BAR in the alternative model 3.5		18 s^{-1}	Estimated

Supplementary Notes

Supplementary Note 1. Model equations and parameters

The model parameters are listed in Supplementary Table 1 and Supplementary Table 2. In the following, we describe the formulation of model equations. The model describes rhythmic propagation emerging from the interplay between membrane-bound chemical reactions and membrane shape changes. We focused on the simplest mechanism and only included the most essential components suggested by experiments. These components are: Cdc42, N-WASP, F-BAR, actin, Arp2/3, and membrane shape. Below, we will translate this qualitative picture into a set of coupled partial differential equations (PDEs). We described the dynamics of all the cortical proteins by reaction-diffusion type equations (see Eqs. (1.1)-(1.5)). All the chemical reactions were modeled by Michaelis-Menten type kinetics, in which the local membrane shape influenced the on rate of F-BAR. Conversely, the local F-BAR level sculptured the shape of the local membrane, and the local actin level modulated the membrane tension. Here, the membrane shape change is captured by a Langevin equation that stems from the functional derivative of membrane mechanical energy with regard to the membrane height variation (see Eqs. (1.6)-(1.8)). In this way, the membrane mechanics was coupled with the local chemical reactions. These PDEs thus depict the spatial-temporal dynamics of the mechanochemical coupling of the membrane shape and the membrane-bound chemical reactions.

<u>*Cdc42 dynamics:*</u>

$$\frac{\partial C}{\partial t} = \underbrace{D_C \cdot \nabla^2 C}_{\text{Cdc42 diffusion}} + \left(\underbrace{s}_{\text{Activation signal}} + \underbrace{k_{on}^{C} \cdot (W + F)}_{\substack{\text{Cortical recruitment of Cdc42} \\ \text{by N-WASP and F-BAR}}} \right) \cdot (1 - C) - \underbrace{k_{off}^{C} \cdot C}_{\text{Cdc42 turnover}} \tag{1.1}$$

Note that the activation signal, *s*, is local and transient (see Fig. 1b).

<u>*N-WASP dynamics:*</u>

$$\frac{\partial W}{\partial t} = \underbrace{D_W \cdot \nabla^2 W}_{\text{N-WASP diffusion}} + \underbrace{k_{on}^{W} \cdot (C + F) \cdot (1 - W)}_{\substack{\text{Cortical recruitment of N-WASP} \\ \text{by Cdc42 and F-BAR}}} - \underbrace{k_{off}^{W} \cdot W}_{\text{N-WASP turnover}} \tag{1.2}$$

<u>*F-BAR dynamics:*</u>

$$\frac{\partial F}{\partial t} = \underbrace{D_F \cdot \nabla^2 F}_{\text{F-BAR diffusion}} + \left(\underbrace{k_{on}^F \cdot (C+W)}_{\substack{\text{Cortical recruitment of F-BAR} \\ \text{by Cdc42 and N-WASP}}} + \underbrace{k_{on}^{Fh} \cdot \left(e^{\left(\nabla^2 h/\Omega_0\right)} - 1 \right)}_{\substack{\text{Membrane curvature induced} \\ \text{F-BAR cortical recruitment}}} \right) \cdot (1-F) - \underbrace{k_{off}^F \cdot F}_{\text{F-BAR turnover}} \tag{1.3}$$

Here, the term $k_{on}^{Fh} \cdot \left(e^{\left(\nabla^2 h/\Omega_0\right)} - 1 \right)$ depicted the membrane curvature-dependence of F-BAR cortical recruitment [8, 16, 35]. h is the local membrane height, and $\nabla^2 h$ is the corresponding membrane curvature (see Eqs. (1.6) – (1.8) for details). Ω_0 is the characteristic membrane curvature, beyond which F-BAR cortical recruitment was significantly increased. We would like to emphasize that the F-BAR proteins in the model refers to a collective functional entity of multiple F-BAR domain proteins including FBP17 and CIP4. This is consistent with our double knockdown experiment results in Fig. 3b. Consequently, the characteristic membrane curvature Ω_0 is within the range of the values measured/inferred from different F-BAR proteins. An *in vitro* experiment demonstrated that the curvature preference of FCHo (an F-BAR protein), was roughly the curvature of a lipid vesicle ~ 200-400 nm in diameter [8]. In the model, we thus chose the nominal value of Ω_0 to be ~ -1/200 nm^{-1}; the negative sign here reflects the fact that F-BAR proteins prefer the membrane bending toward the cell interior. Our phase diagram studies showed that traveling waves can form over a broad range of this threshold curvature Ω_0 (Fig. 3a).

In principle, the proteins could also surf along hydrodynamic flow of lipids within the bilayer; such a curvature-driven motion would be a real material propagation in the direction of wave propagation. How much this mode of motion contributes to the wave propagation in part depends on how long the proteins remain bound to the membrane. Experiments show that F-BAR dissociates from membrane rapidly with a lifetime on the order of seconds (for instance, see ref. [16]). This is also consistent with our experimental observation that the $t_{1/2}$ of F-BAR puncta is ~ a few seconds during the wave propagation (Fig. 5g). In contrast, single waves typically propagate for ~ 20 seconds or longer in our experiments. This suggests that the wave does not consist of individual proteins surfing in the direction of wave propagation over long timescales. We therefore assumed in our current model that this mode of protein motion was not significant. This is consistent with our zoom-in kymograph (Fig. 5g), which shows that F-BAR puncta do not move notably in the direction of the wave propagation. Also, our model treated the membrane as an elastic sheet, which by its nature is incapable of describing hydrodynamic in-plane flow of lipids within the bilayer. In the future, we would like to systematically investigate how the in-plane lipid flow might influence traveling waves in a more general setting.

Arp2/3 dynamics:

$$\frac{\partial R}{\partial t} = \underbrace{D_R \cdot \nabla^2 R}_{\text{Arp2/3 diffusion}} + \underbrace{\left(k_{on}^R \cdot \frac{\left(W / W_0\right)^2}{1+\left(W / W_0\right)^2} \right) \cdot (1-R)}_{\text{N-WASP-dependent Arp2/3 activation}} - \underbrace{k_{off}^R \cdot R}_{\text{Arp2/3 inactivation}} \tag{1.4}$$

Note that N-WASP is known to activate Arp2/3 complex as a dimer [36]. Therefore, the Hill coefficient is 2.

Actin dynamics:

$$\frac{\partial A}{\partial t} = \underbrace{\left(k_{on}^A \cdot \frac{\left(R / R_0\right)^4}{1+\left(R / R_0\right)^4} \right) \cdot (1-A)}_{\text{Arp2/3 mediated actin polymerization}} - \underbrace{k_{off}^A \cdot A}_{\text{Actin depolymerization}} \tag{1.5}$$

While many proteins are at play, the Eq. (1.5) with Hill coefficient = 4 is the simplest form that is meant to capture this nonlinearity. Any cooperativity with Hill coefficient ≥ 2 preserves the model predictions concerning traveling waves (Supplementary Fig. 11e). We note that Arp2/3-mediated branched actin polymerization is autocatalytic. It involves nucleation phase with latency followed by rapid actin polymerization and, hence, is highly non-linear [37]. For instance, branched actin network polymerization itself could provide more F-actin plus ends, which could locally accumulate more Arp2/3-complex [38]. Alternatively, there are many other proteins could feed back with Arp2/3-mediated actin polymerization. For example, WIP is another molecule involved in the axis of N-WASP – Arp2/3 – branching actin polymerization: WIP is activated by N-WASP, and subsequently facilitates Arp2/3-mediated actin polymerization [39]. Another actin nucleation factor, formin, could also participate in Arp2/3-mediated actin polymerization [40]. In all cases, the Hill coefficient is expected to be higher than 2. The combination of Eqs. (1.2), (1.4) and (1.5) phenomenologically describes the nonlinear dynamics of actin polymerization with an effective time delay that lags N-WASP activation.

Membrane shape dynamics:

$$E\left(h(x,y)\right) = \iint dx\,dy \left(\underbrace{\sigma_m \left(\nabla h\right)^2}_{\text{Surface tension}} + \underbrace{\kappa_m \left(\nabla^2 h - \underbrace{\Omega_F F}_{\text{Preferred curvature by F-BAR}} \right)^2}_{\text{Bending energy}} + \underbrace{\frac{1}{2} \tilde{k}_0 h^2}_{\text{Adhesion energy to the substrate}} \right) \tag{1.6}$$

$$\lambda_m \cdot \frac{\partial h}{\partial t} = -\frac{\delta E}{\delta h}$$
$$= 2\sigma_m \nabla^2 h - 2\kappa_m \nabla^4 h + 2\kappa_m \Omega_F \nabla^2 F - \kappa_m \Omega_F^2 F \frac{\delta F}{\delta h} - \tilde{k}_0 h$$
$$= 2\sigma_m \nabla^2 h - 2\kappa_m \nabla^4 h + 2\kappa_m \Omega_F \nabla^2 F + f_F F - \tilde{k}_0 h \tag{1.7}$$

$$\sigma_m = \sigma_0 \cdot \left(1 + \alpha_A \cdot \frac{\left(A / A_0\right)^3}{1 + \left(A / A_0\right)^3} \right) \tag{1.8}$$

Eq. (1.6) describes membrane as an elastic sheet, whose mechanical energy is depicted by the Helfrich membrane free energy *E*. *h* is the instantaneous local membrane height in the z-direction under Monge Gauge (*i.e.*, *h* is very small compared to the membrane patch size in the x and y directions. $\nabla^2 h$ is the local mean curvature, and the preferred curvature by F-BAR, $\Omega_F F$, is linearly proportional to the local F-BAR level, *F*. κ_m is the bending modulus and σ_m is the surface tension. $\frac{1}{2}\tilde{k}_0 h^2$ is the adhesion energy between the membrane and the substrate, given that our traveling waves are on the ventral membrane of the cell.

Eq. (1.7) is a Langevin equation that describes the dynamics of membrane shape changes. It stems from the functional derivative of the free energy functional with regard to the variation of the membrane height *h*. λ_m is effective viscous drag coefficient for membrane shape changes; it combines the membrane resistance and fluid drag from outside, whose value is measured to be ~ 2×10^9 Pa s m^{-1} [7]. In addition, the functional derivative $\frac{\delta F}{\delta h}$ at the membrane equals to the derivative of the F-BAR concentration field in the normal direction of the membrane surface. The F-BAR concentration in this direction has a gradient low in cytoplasm and high at the plasma membrane, so it is negative. In the simplest approximation, we assumed that this gradient at the membrane is a constant $-g_F$ with $g_F > 0$. Combined together, $\kappa_m \Omega^2{}_F F \frac{\delta F}{\delta h} = -f_F F$. Here, $f_F = g_F \kappa_m \Omega^2{}_F$, it is essentially the free energy change arising from F-BAR binding to membrane (similar to chemical potential), from which the value of f_F can be estimated. Furthermore, the F-BAR expression level under this study is low; otherwise, significant membrane tubulation would be evident. In the low F-BAR level limit, the linear term in F always dominates over $\nabla^2 F$ term in Eq. (1.7). In our calculation, we thus ignored the $\nabla^2 F$ term.

Eq. (1.8) characterizes the modulation of membrane tension by local cortical actin level. Actin binding to membrane is a complex process and supposed to be highly cooperative. For example, experiments in *dictyostelium discoideum* showed that actin binds to membrane with Hill coefficient = 3 [41]. We therefore used the Hill coefficient = 3 in our model. The qualitative feature of our model remains the same for any hill coefficient > 2 (data not shown). It also has been measured that cortical actin polymerization could

increase the measured plasma membrane tension by 2-10 fold [28, 29, 30, 31], which sets the range of α_A in Eq. (1.8).

Computational scheme

In the simulation, a clamped boundary condition was imposed. The initial condition was a flat membrane on which all the cortical protein concentrations are zero. At time zero, an activation signal was imposed to the system. We solved the coupled PDEs by the finite element solver COMSOL® Multiphysics software (version 4.4). The spatial resolution is on the order of 300 nm. All the related parameters are rescaled according to this setup. The time dependent solver was used with relative tolerance of $1\mathrm{x}10^{-4}$. The backward differentiation formula or BDF method was used for time stepping provided by default Multiphysics module of COMSOL. The solver used is MUMPS (multifrontal massively parallel sparse direct solver) from default Multiphysics module of COMSOL.

Boundary conditions

We implemented the clamped boundary condition by an adhesive stress term. We assumed that this adhesive stress stems from the overall effect of focal adhesions, which adhere and, hence, clamp the membrane at the leading edge to the substrate. This effect is different from that of the generic membrane-substrate adhesion (*i.e.*, the last term in Eq. (1.6)), which is believed to largely depend on hydration forces and charge-charge interactions between the membrane and the substrate. Because focal adhesion density decreases from the leading edge to the interior (Fouchard *et al.* [42] and Möhl *et al.* [43]), we implemented the clamped boundary condition in accordance with this spatial dependence (see Supplementary Fig. 12a). Here, we chose the form of the adhesive stress as $f_{Clamp} = f_c \cdot h / h_{\max}$, where $f_c = f_{c0} \cdot \dfrac{\left(\left(r-(R-2r_0)\right)/r_0\right)^n}{1+\left(\left(r-(R-2r_0)\right)/r_0\right)^n}$, r is the distance from the epicenter, and R is the radius of the simulation domain. $2r_0$ is the width of the region where this adhesive stress is applied (~ 1 – 6 μm [42, 43], we used 2 μm in the model). Consequently, $r = R\text{-}2r_0$ is where the boundary adhesive stress starts to take effect. f_{c0} is the maximum adhesive stress due to the adhesions near the cell edge (~ 0.4 – 9 kPa [44, 45, 46], we used 1 kPa in the model). Additionally, $h(r)$ is the local membrane height. h_{max} is the maximum membrane height from the substrate, which can be inferred from the range of integrin-substrate deformation measured by Jiang *et al.* [45] (~ 200 – 1000 nm). We thus chose the value of $h_{\max}$ to be 300 nm in the model. We note that our model conclusions are largely insensitive to the variations of these model parameters. Below, we further explain the derivation of relevant model parameters from experimental data.

f_{c0}: The experimental work by Gallant *et al.* [44] shows that the equilibrium normal tensile force between focal adhesions and the substrate ranges from ~ 180 nN – 220 nN with corresponding adhesive area ~19.6 – 314 μm^2. This gives the maximum adhesive stress of ~ 0.6 – 9 kPa for stable adhesive patch. On the other hand, single molecule force measurements by Jiang *et al.* [45] show that the bond between integrin and substrate breaks

at ~ 1 – 3 pN. Given that the density of integrins within focal adhesions is ~400 – 1000 μm^{-2} [46], this yields the maximum adhesive stress of ~ 0.4 – 3 kPa. Taken together, the maximum adhesive stress is ~ 0.4 – 9 kPa. We chose the value of f_{c0} to be 1 kPa in the model. The model predictions are not sensitive to the choice of f_{c0}.

$2r_0$ and *n*: The experiments by Fouchard *et al.* [42] and Möhl *et al.* [43] show that the average density of focal adhesion molecules (*e.g.*, paxillin and vinculin) sharply decreases to background levels from its maxium value within ~ 1 – 6 μm (Fig 1 in [43] and Fig. S2 in [42]). We chose $2r_0$ to be 2 μm in the model, which accordingly takes the location of the maximum adhesion stress as the exact boundary in our simulation. Further, fitting the experimental data (Fig. S2 in [42]) to a sigmoidal function suggested that the corresponding Hill coefficient in ($\frac{\left(\left(r-(R-2r_0)\right)/r_0\right)^n}{1+\left(\left(r-(R-2r_0)\right)/r_0\right)^n}$) should be ~ 2 – 6. We used 4 in the model. The model predictions are not sensitive to the choices of $2r_0$ and the Hill coefficient, n.

Supplementary Note 2. Analytic solution of traveling wave

To understand the nature of the traveling wave more precisely, we derive the analytical expression of traveling wave speed through 3 simplification steps.

Simplification 1:

In the full system presented in this work (Eqs. (1.1)-(1.8)), the cortical proteins Cdc42, N-WASP and F-BAR (CWF) work together as a functional module. Although the downstream pathways are regulated by different parts of the module, it is reasonable to simplify the system by treating the CWF module as a single entity, Z. We assume that the reaction rates of *C* and *W* are fast and C and W can be replaced by their quasi-steady state values. This simplification is valid because the reaction rates within the CWF module are faster than the other chemical reactions, as supported by the model parameters (see Supplementary Table 1 and Supplementary Table 2). Therefore, the number of equations is decreased to 4 as shown below.

$$\frac{\partial Z}{\partial t} = D_Z \cdot \nabla^2 Z + \left[s + k_{on}^{Z} \cdot Z + k_{on}^{Zh} \cdot \left(e^{\frac{\nabla^2 h}{\Omega_0}} - 1 \right) \right] \cdot (1 - Z) - k_{off}^{Z} \cdot Z$$
$$\approx D_Z \cdot \nabla^2 Z + \left[s + k_{on}^{Z} \cdot Z + \frac{k_{on}^{Zh}}{\Omega_0} \cdot \nabla^2 h \right] \cdot (1 - Z) - k_{off}^{Z} \cdot Z \tag{2.1}$$

$$\frac{\partial R}{\partial t} = D_R \cdot \nabla^2 R + \left[k_{on}^{R} \cdot \frac{(Z/Z_1)^2}{1 + (Z/Z_1)^2} \right] \cdot (1 - R) - k_{off}^{R} \cdot R \tag{2.2}$$

$$\frac{\partial A}{\partial t} = \left[k_{on}^{A} \cdot \frac{(R/R_0)^4}{1 + (R/R_0)^4} \right] \cdot (1 - A) - k_{off}^{A} \cdot A \tag{2.3}$$

$$\frac{\partial h}{\partial t} = \frac{1}{\lambda_m} \cdot \left[2\sigma_0 \left(1 + \alpha_A \frac{(A/A_0)^3}{1 + (A/A_0)^3} \right) \cdot \nabla^2 h - 2\kappa_m \nabla^4 h + 2\kappa_m \Omega_F \nabla^2 Z + f_F \cdot Z - \tilde{k}_0 \cdot h \right] \tag{2.4}$$

Here, the approximation in Eq. (2.1) assumes $\nabla^2 h << 1$. The term $k_{on}^{Z} \cdot Z$ represents the simplest form of self-promotion of the CWF module, which qualitatively matches with the C-W-F pathway behavior in the full system. To have a better correspondence with the original system, we estimated the relationship between k_{on}^{Z} and the other original rate constants (see model parameter Supplementary Table 1 and Supplementary Table 2). Assuming that *C* and *W* are at quasi-steady state, from Eqs (1.1)-(1.3), we have

$$C \approx \frac{s + k_{on}^{C} \cdot (W + F)}{k_{off}^{C} + k_{on}^{C}(W + F) + s} \tag{2.5}$$

$$W \approx \frac{k_{on}^{W} \cdot (C + F)}{k_{off}^{W} + k_{on}^{W} \cdot (C + F)} \tag{2.6}$$

For the system of C and W without activation, we have the solutions for C and W as the function of F as follows:

$$C = \frac{-(K_{C3} + K_{C4}F + K_{C2}F^2) + \sqrt{(K_{C3} + K_{C4}F + K_{C2}F^2)^2 + 4(K_{C1} + K_{C2}F)\cdot(K_{C5}F + K_{C2}F^2)}}{2(K_{C1} + K_{C2}F)} \tag{2.7}$$

$$W = \frac{-(K_{W3} + K_{W4}F + K_{W2}F^2) + \sqrt{(K_{W3} + K_{W4}F + K_{W2}F^2)^2 + 4(K_{W1} + K_{W2}F)\cdot(K_{W5}F + K_{W2}F^2)}}{2(K_{W1} + K_{W2}F)} \tag{2.8}$$

where

$$K_{C1} = k_{on}^{W} \cdot \left(k_{on}^{C} + k_{off}^{C}\right); K_{C2} = k_{on}^{C} \cdot k_{on}^{W}; K_{C3} = k_{off}^{C} \cdot k_{off}^{W} - k_{on}^{C} k_{on}^{W}$$

$$K_{C4} = k_{on}^{W} k_{off}^{C} + k_{off}^{W} k_{on}^{C}; K_{C5} = k_{on}^{C} \cdot \left(k_{on}^{W} + k_{off}^{W}\right)$$

$$K_{W1} = k_{on}^{C} \cdot \left(k_{on}^{W} + k_{off}^{W}\right); K_{W2} = k_{on}^{W} \cdot k_{on}^{C}; K_{W3} = k_{off}^{C} \cdot k_{off}^{W} - k_{on}^{W} k_{on}^{C}$$

$$K_{W4} = k_{on}^{W} k_{off}^{C} + k_{off}^{C} k_{on}^{W}; K_{W5} = k_{on}^{W} \cdot \left(k_{on}^{C} + k_{off}^{C}\right)$$

Then the rate term in Eq. (1.3) can be approximately written in the limit of F<<1.

$$\begin{aligned}
k_{on}^{F} \cdot (C + W) &\approx k_{on}^{F} \cdot \left(\frac{\frac{2K_{C1}K_{C5}}{K_{C3}}F - \frac{1}{2}K_{C4}F}{K_{C1} + K_{C2}F} + \frac{\frac{2K_{W1}K_{W5}}{K_{W3}}F - \frac{1}{2}K_{W4}F}{K_{W1} + K_{W2}F} \right) \\
&\approx k_{on}^{F} \cdot \left(\frac{2K_{C5}}{K_{C3}} - \frac{K_{C4}}{2K_{C1}} + \frac{2K_{W5}}{K_{W3}} - \frac{K_{W4}}{2K_{W1}} \right) F \\
&\approx k_{on}^{Z} \cdot Z
\end{aligned} \tag{2.9}$$

This simplified estimation can be used as a guideline for choosing the Z-related reaction rate parameters. Based on parameter table for the full model, k_{on}^{Z} in Eq. (2.9) will be ~ several to 10's times larger than k_{on}^{F}.

Simplification 2:

In the full model, CWF (we denote as "*Z*" here) promotes Arp2/3 (denoted as "*R*") that in turn drives actin polymerization (denoted as "*A*"). The resulting F-actin level stiffens the

membrane tension via a highly nonlinear sigmoidal response curve. If we assume that the dynamics of Arp2/3 activation and actin polymerization are fast and at quasi-equilibrium state, we could solve the Eqs. (2.2) and (2.3) by ignoring the diffusion term in *R*. Consequently, the actin level in Eq. (1.8) could be represented as a function of *Z*. To keep the essence of the nonlinear ultrasensitive response curve in Eq. (1.8) but simplify the math, we treated the $Z \rightarrow R \rightarrow A \rightarrow h$ pathway as one Michaelis-Menten kinetics process for *Z* in the PDE for the dynamics of membrane height. The model is then only characterized by the dynamics of *h* and *Z* – Eqs (2.10) and (2.11). In Eq. (2.10), $N \sim 12$ as the combined Hill coefficient from Eqs (2.2), (2.3) and (1.8).

$$\frac{\partial h}{\partial t} = \frac{1}{\lambda_m}\left[2\sigma_0\left(1+\alpha_A\frac{(Z/Z_0)^N}{1+(Z/Z_0)^N}\right)\nabla^2 h - 2\kappa_m\nabla^4 h + 2\kappa_m\Omega_F\nabla^2 Z + f_F Z - \tilde{k}_0 h\right] \tag{2.10}$$

$$\frac{\partial Z}{\partial t} = D_Z\nabla^2 Z + \left[s + k_{on}^Z Z + k_{on}^{Zh}\left(e^{\frac{\nabla^2 h}{\Omega_0}} - 1\right)\right](1-Z) - k_{off}^Z Z \tag{2.11}$$

Simplification 3:

To further simplify Eq. (2.11), we ignore the diffusion term. This simplification assumes that the origin of spatial variation in *Z* fully follows that of the membrane curvature $\nabla^2 h$, consistent with our numerical calculation of the full model and experimental observation, such as those shown in Figs. 2-5 in the main text. We thus obtain Eq. (2.12).

$$\frac{\partial Z}{\partial t} = \left[s + k_{on}^Z Z + k_{on}^{Zh}\left(e^{\frac{\nabla^2 h}{\Omega_0}} - 1\right)\right](1-Z) - k_{off}^Z Z \tag{2.12}$$

In the limit of very small membrane curvature (*i.e.*, $\nabla^2 h \ll 1$), we expand the exponential term in Eq. (2.12) and keep the linear and 2nd order term in $\nabla^2 h$. Consequently, we obtain Eq. (2.13):

$$\frac{\partial Z}{\partial t} = \left[s + k_{on}^Z Z + \frac{k_{on}^{Zh}}{\Omega_0}\nabla^2 h + \frac{k_{on}^{Zh}}{2\Omega_0^{\;2}}(\nabla^2 h)^2 + O\left((\nabla^2 h)^3\right)\right](1-Z) - k_{off}^Z Z \tag{2.13}$$

Analytic solution of traveling wave:

In seeking traveling wave solution, we assume that the dynamics of Z is faster than the membrane height h such that Z reaches chemical equilibrium faster. With $\partial Z/\partial t \sim 0$, we obtained Z as the function of the membrane curvature.

$$-k_{on}^{Z}Z^2+\left(k_{on}^{Z}-\left(k_{off}^{Z}+s+\frac{k_{on}^{Zh}}{\Omega_0}\nabla^2 h+\frac{k_{on}^{Zh}}{2\Omega_0^2}\left(\nabla^2 h\right)^2\right)\right)Z+\left(s+\frac{k_{on}^{Zh}}{\Omega_0}\nabla^2 h+\frac{k_{on}^{Zh}}{2\Omega_0^2}\left(\nabla^2 h\right)^2\right)=0 \tag{2.14}$$

The positive solution for Z up to second order terms in $\nabla^2 h$ is:

$$Z=\frac{k_{on}^{Z}-k_{off}^{Z}-s+\left(\left(s-k_{on}^{Z}+k_{off}^{Z}\right)^2+4k_{on}^{Z}s\right)^{1/2}}{2k_{on}^{Z}}+\frac{1}{2k_{on}^{Z}}\left(\frac{\left(s+k_{on}^{Z}+k_{off}^{Z}\right)\frac{k_{on}^{Zh}}{\Omega_0}}{\left(\left(s-k_{on}^{Z}+k_{off}^{Z}\right)^2+4k_{on}^{Z}s\right)^{1/2}}-\frac{k_{on}^{Zh}}{\Omega_0}\right)\nabla^2 h$$
$$+\frac{1}{2k_{on}^{Z}}\left(\frac{\left(\frac{k_{on}^{Zh}}{\Omega_0}\right)^2+\left(s+k_{on}^{Z}+k_{off}^{Z}\right)\frac{k_{on}^{Zh}}{\Omega_0^2}}{2\left(\left(s-k_{on}^{Z}+k_{off}^{Z}\right)^2+4k_{on}^{Z}s\right)^{1/2}}-\frac{\left(s+k_{on}^{Z}+k_{off}^{Z}\right)^2\left(\frac{k_{on}^{Zh}}{\Omega_0}\right)^2}{2\left(\left(s-k_{on}^{Z}+k_{off}^{Z}\right)^2+4k_{on}^{Z}s\right)^{3/2}}-\frac{k_{on}^{Zh}}{2\Omega_0^2}\right)\left(\nabla^2 h\right)^2 \tag{2.15}$$

Now plugging Eq. (2.15) back to Eq. (2.10), we obtain the single equation (Eq. (2.16)) in terms of the membrane height h and the membrane curvature $\nabla^2 h$, which describes the nonlinear dynamics of membrane shape changes in the model. Here we ignore the bending energy contribution (the $\nabla^4 h$ term), because the lateral dimension of the membrane shape deformation is in the range of microns, over which the driving force from bending energy is much smaller than that from membrane tension. We also ignore the $\nabla^2 Z$ term as it gives to $\nabla^4 h$ and $\nabla^6 h$ terms, which are much smaller than $\nabla^2 h$ in the limit of $\nabla^2 h << 1$.

$$\frac{\partial h}{\partial t}=\frac{f_F}{\lambda_m}b_1-\frac{\tilde{k}_0}{\lambda_m}h+\left(\frac{2\sigma_0}{\lambda_m}+\frac{2\alpha_A\sigma_0}{\lambda_m}\frac{\left(b_1/Z_0\right)^N}{1+\left(b_1/Z_0\right)^N}+\frac{f_F}{\lambda_m}b_2\right)\nabla^2 h$$
$$+\left(\frac{2\alpha_A\sigma_0}{\lambda_m}\cdot\frac{Nb_2}{Z_0}\cdot\frac{\left(b_1/Z_0\right)^{N-1}}{\left(1+\left(b_1/Z_0\right)^N\right)^2}+\frac{f_F}{\lambda_m}b_3\right)\left(\nabla^2 h\right)^2 \tag{2.16}$$

where

$$b_1=\frac{k_{on}^{Z}-k_{off}^{Z}-s+\left(\left(s-k_{on}^{Z}+k_{off}^{Z}\right)^2+4k_{on}^{Z}s\right)^{1/2}}{2k_{on}^{Z}}$$

$$b_2 = \frac{1}{2k_{on}^Z}\left(\frac{\left(s + k_{on}^Z + k_{off}^Z\right)\frac{k_{on}^{Zh}}{\Omega_0}}{\left(\left(s - k_{on}^Z + k_{off}^Z\right)^2 + 4k_{on}^Z s\right)^{1/2}} - \frac{k_{on}^{Zh}}{\Omega_0} \right)$$

$$b_3 = \frac{1}{2k_{on}^Z}\left(\frac{\left(\frac{k_{on}^{Zh}}{\Omega_0}\right)^2 + \left(s + k_{on}^Z + k_{off}^Z\right)\frac{k_{on}^{Zh}}{\Omega_0^2}}{2\left(\left(s - k_{on}^Z + k_{off}^Z\right)^2 + 4k_{on}^Z s\right)^{1/2}} - \frac{\left(s + k_{on}^Z + k_{off}^Z\right)^2\left(\frac{k_{on}^{Zh}}{\Omega_0}\right)^2}{2\left(\left(s - k_{on}^Z + k_{off}^Z\right)^2 + 4k_{on}^Z s\right)^{3/2}} - \frac{k_{on}^{Zh}}{2\Omega_0^2} \right)$$

Taking Laplacian for both sides of Eq. (2.16), we define $H=\nabla^2 h$. We then have:

$$\begin{aligned} \frac{\partial H}{\partial t} &= -\frac{\tilde{k}_0}{\lambda_m}H + \left(\frac{2\sigma_0}{\lambda_m} + \frac{2\alpha_A\sigma_0}{\lambda_m}\frac{\left(b_1 / Z_0\right)^N}{1+\left(b_1 / Z_0\right)^N} + \frac{f_F}{\lambda_m}b_2 \right)\nabla^2 H \\ &+ \left(\frac{2\alpha_A\sigma_0}{\lambda_m}\cdot\frac{Nb_2}{Z_0}\cdot\frac{\left(b_1 / Z_0\right)^{N-1}}{\left(1+\left(b_1 / Z_0\right)^N\right)^2} + \frac{f_F}{\lambda_m}b_3 \right)\nabla^2\left(H^2\right) \end{aligned} \qquad (2.17)$$

We further drop the linear term of H in the Eq. (2.17). This is because according to the model parameters (Supplementary Table 1 and Supplementary Table 2), the membrane-substrate adhesion energy term is typically orders-of-magnitude smaller than the rest of the terms in Eq. (2.17). We thus arrive at the final PDE from which we seek traveling wave solutions.

$$\frac{\partial H}{\partial t} = \left(\frac{2\sigma_0}{\lambda_m} + \frac{2\alpha_A\sigma_0}{\lambda_m}\frac{\left(b_1 / Z_0\right)^N}{1+\left(b_1 / Z_0\right)^N} + \frac{f_F}{\lambda_m}b_2 \right)\nabla^2 H + \left(\frac{2\alpha_A\sigma_0}{\lambda_m}\cdot\frac{Nb_2}{Z_0}\cdot\frac{\left(b_1 / Z_0\right)^{N-1}}{\left(1+\left(b_1 / Z_0\right)^N\right)^2} + \frac{f_F}{\lambda_m}b_3 \right)\nabla^2\left(H^2\right) \qquad (2.18)$$

To seek "steady-state" solutions for a traveling wave, we transform to a coordinate system moving with constant speed V via the coordinate transformation $\xi = x\text{-}Vt$ and $H = H(\xi)$. In this coordinate system the activation signal, s, in the b_1, b_2, and b_3-terms is zero. We focused on the simplest case, in which the wave only propagates in one direction. The membrane shape thus varies only in x-direction. The Eq. (2.18) became:

$$\left(H + \frac{B_2}{2B_1} \right)H'' + \left(H'\right)^2 + \frac{V}{2B_1}H' = 0 \qquad (2.19)$$

where

$$B_1 = \frac{2\alpha_A\sigma_0 b_2 N}{\lambda_m Z_0}\cdot\frac{\left(b_1 / Z_0\right)^{N-1}}{\left(1+\left(b_1 / Z_0\right)^N\right)^2} + \frac{f_F}{\lambda_m}b_3$$

$$B_2 = \frac{2\sigma_0}{\lambda_m} + \frac{2\alpha_A\sigma_0}{\lambda_m}\frac{(b_1/Z_0)^N}{1+(b_1/Z_0)^N} + \frac{f_F}{\lambda_m}b_2$$

Re-defining the function:

$$Q = H + \frac{B_2}{2B_1} \ ,$$

we arrive at Eq. (3.20):

$$0 = QQ'' + (Q')^2 + \frac{V}{2B_1}Q' \tag{2.20}$$

This leads to:

$$QQ' = -\beta Q + \gamma \tag{2.21}$$

where γ is a constant.

The first solution of Eq. (2.21) is Q=constant and Q'=0. With the boundary condition for traveling wave as $H(\xi) \to 0$ and $h(\xi) \to 0$ for $\xi \to \infty$, this implies that if H is a constant, then it must be zero to meet this boundary condition. As such, h=0 for the entire space – a flat membrane. Therefore, this solution is trivial.

Now, with $Q' \neq 0$, we have the second solution:

$$e^{Q(\xi)-Q(0)}\left(\frac{Q(\xi)-\frac{\gamma}{\beta}}{Q(0)-\frac{\gamma}{\beta}}\right)^{\frac{\gamma}{\beta}} = e^{-\beta\xi} \text{ and } \beta = \frac{V}{2B_1}$$

The requirement of $H(\xi) \to 0$ for $\xi \to \infty$ entails that

$$\frac{\gamma}{\beta} = \frac{B_2}{2B_1} \tag{2.22}$$

Therefore the solution reduces to:

$$e^{\nabla^2 h(\xi)-\nabla^2 h(0)}\left(\frac{\nabla^2 h(\xi)}{\nabla^2 h(0)}\right)^{\frac{\gamma}{\beta}} = e^{-\beta\xi} \tag{2.23}$$

Note that Eq. (2.23) is valid under the condition of nonzero membrane curvature at the wavefront, *i.e.*, $\nabla^2 h(0) \neq 0$. In the limit of $\xi \to 0^+$, *i.e.*, near the wave front, $\nabla^2 h(\xi) = \nabla^2 h(0) + \xi\left(\nabla^2 h(0)\right)'$. Eq. (2.23) yields traveling wave speed as:

$$V = \left(\nabla^2 h(0)\right)' \cdot \left\{\frac{1}{\left|\nabla^2 h(0)\right|} \cdot \left[\frac{2\sigma_0}{\lambda_m} + \frac{2\alpha_A\sigma_0}{\lambda_m}\frac{(b_1/Z_0)^N}{1+(b_1/Z_0)^N} + \frac{f_F}{\lambda_m}b_2\right] - 2\left[\frac{2\alpha_A\sigma_0 b_2 N}{\lambda_m Z_0} \cdot \frac{(b_1/Z_0)^{N-1}}{\left(1+(b_1/Z_0)^N\right)^2} + \frac{f_F}{\lambda_m}b_3\right]\right\}$$

This formula leads to the Eq. (2.24):

$$V = \underbrace{\frac{\left(\nabla^2 h(0)\right)'}{\left|\nabla^2 h(0)\right|}\cdot\frac{2\sigma_0}{\lambda_m}}_{\text{Inherent membrane mechanics}} + \underbrace{\frac{f_F}{\lambda_m}\cdot\frac{\left(\nabla^2 h(0)\right)'}{k_{off}^Z - k_{on}^Z}\cdot\left[\frac{k_{on}^{Zh}}{\Omega_0}\cdot\frac{\Omega_0 - \left|\nabla^2 h(0)\right|}{\left|\nabla^2 h(0)\right|\Omega_0} + 4\left(\frac{k_{on}^{Zh}}{\Omega_0}\right)^2\cdot\frac{k_{off}^Z}{\left(k_{off}^Z - k_{on}^Z\right)^2}\right]}_{\text{Curvature sensing}} \tag{2.24}$$

At the wave front, the membrane curvature is the most negative, so its local gradient of the membrane curvature is positive. Again, we must emphasize that the derivation of the traveling wave solution dictates that Eq. (2.24) is only valid for the nonzero curvature at the wave front ($\nabla^2 h(0) \neq 0$), which prevents the divergence of this wave speed from this formula. With this constraint, Eq. (2.24) suggests that while the wave speed increases with the gradient of curvature at the wavefront, it decreases with the membrane curvature at the wavefront; *i.e.,* the smaller the membrane curvature at the wavefront $\left|\nabla^2 h(0)\right|$, the faster the traveling wave velocity *V*. In other words, the flatter the membrane at the wave front is, the faster the wave travels. We note that the traveling wave of our system has both a mechanical part and chemical part, *i.e.*, the membrane wave couples with the wave of cortical protein concentration. Eq. (2.24) characterizes the wave speed both for the chemical wave and its accompanying membrane wave.

An important note is that while Eq. (2.24) describes the relationship between wave speed, membrane shape, membrane mechanics, and cortical reaction rates, one cannot use it to directly calculate the steady-state wave speed. This is because the membrane shape variable in Eq. (2.24) is a model output, which itself is determined by the mechanochemistry of the system, and thus cannot be assumed *a priori*. In this sense, the wave speed could depend on the membrane mechanics and cortical reaction rates in a convoluted manner. For instance, increasing membrane tension σ_0 in Eq. (2.24) will flatten the membrane at wave front. While both $\left|\nabla^2 h(0)\right|$ and $\left(\nabla^2 h(0)\right)'$ should decrease in this case, it could be that $\left(\nabla^2 h(0)\right)'$ decreases more than $\left|\nabla^2 h(0)\right|$. In that case, the change in $\frac{2\sigma_0}{\lambda_m}$ would compensate the change in $\frac{\left(\nabla^2 h(0)\right)'}{\left|\nabla^2 h(0)\right|}$ and there would be little effect on wave speed. This might explain the observed insensitivity of wave speed to membrane tension (Fig. 4d).

<u>*Estimation of upper limit of wave speed:*</u>

We next used this formula to estimate the upper limit of wave speed. To that end, we only need to estimate the lowest value of $\left|\nabla^2 h(0)\right|$ and the highest value of $\left(\nabla^2 h(0)\right)'$, as the rest of the parameters in Eq. (2.24) are known based on the model parameters given in the parameter tables. Geometrically, the curvature and the curvature gradient could be independent variables. In our system, the lower limit of the membrane curvature is ~ 1/10 μm^{-1}), which relates to the dimension of the cell. To estimate the gradient of the curvature at the wave front, we considered a wave front with the typical lateral dimension ~ 5 μm. It is reasonable to assume that the largest curvature and hence, the variation in curvature in our system are defined by the preferred curvature by F-BAR protein (Ω_0 ~ 1/0.2 μm^{-1}). Combining the wave front size and this largest curvature variation, we estimated that the largest curvature gradient could be ~ 1 μm^{-2}, although irregular membrane shapes could certainly increase this value and, consequently, the wave speed.

As the Eq. (2.24) shows, the upper limit of the wave speed consists of three sources. The first component is the intrinsic membrane tension. The higher the membrane tension is, the faster the wave is. With the inherent membrane tension ~ 10^{-4} N m^{-1}, it alone contributes to the wave speed ~ 1 μm s^{-1}. The second component stems from the membrane stiffening by the cortical proteins. According to the model parameters in Supplementary Table 1 and Supplementary Table 2, this term contributes up to ~ 10-fold increases in the membrane tension, which thus could increase the wave speed by ~ 10 μm s^{-1}. The last contribution arises from the F-BAR protein-mediated membrane deformation, which reduces wave speed due to the preferred curvature by F-BAR (Ω_0 <0). Accordingly, this term reduces the wave speed to ~1 μm s^{-1}. Taken together, the upper speed limit of this wave is ~ 10 μm s^{-1}. We note that this value should only be taken as an order-of-magnitude estimate due to the simplifications required to get the analytical solution.

Comparison between analytic solution and full model result:

After obtaining the analytic solutions, we next studied to what extent these analytic approximations are valid. We compared Eq. (2.24) with the full model numerical results. For faithful comparison, we kept all the relevant model parameters the same between the analytic formula and the full model result.

To compare, we first calculated the full model result that yielded not only the instantaneous wave speed, but also the instantaneous membrane curvature, and its gradient at the wavefront. We then used the wavefront membrane curvature and its gradient from the full model results in the analytic formula to calculate the instantaneous wave speed in our simplified model (Fig. 5a). Fig. 5a shows that the analytic result is consistent, but not identical, with the full model result.

A final note is that due to the complexity of the model, the current analytic solutions could only capture part of the full picture. These analytic solutions are only valid in the limiting case where membrane curvature is small, membrane-substrate adhesion is much smaller than membrane tension, and cortical protein dynamics are faster than membrane shape changes. If there are no actin cortex-mediated modulations, the membrane tension will then reduce significantly, which will render the membrane-substrate adhesion relatively larger and even comparable to the membrane tension. This will invalidate the application of this analytic solution. Consequently, the contribution of the inherent membrane tension to wave speed can only be appropriately interpreted when the cortical proteins are present and the membrane tension is relative high. Similar caution should be taken when the membrane tension is reduced directly by osmolarity or surfactant treatment. More detailed mathematical studies of the model and theoretical analysis will be the subject of future work.

Supplementary Note 3. Alternative model schemes

In this section, we altered the model to further dissect roles of model components in the formation of traveling waves. All the altered models maintained the nature of mechanochemical feedback. Here we focused on the role of curvature sensing in shaping the unusual features of our traveling wave. In particular, we aimed to understand why our wave speed always depended on the protein lateral diffusion constant sub-diffusively.

Below, the first two altered models (3.1 and 3.2) eliminated curvature-sensing effects. While these models are not consistent with our experiments in Fig. 3 that demonstrate~~s~~ the essential role of F-BAR curvature sensitivity for wave formation, we wanted to investigate from a theoretical standpoint what would happen in the absence of curvature-sensing effects. Mathematically, these models are no different from the conventional reaction-diffusion systems. Accordingly, in both models, the wave speed was diffusion-dependent (Supplementary Figs. 11a and b), very similar to conventional chemical waves (*e.g.*, those from BZ reactions). In comparison, the wave speed always is sub-diffusive in our nominal model scheme (Fig. 5) as well as other scenarios (the altered models 3.3-6) (Supplementary Figs. 11c-f) that maintained the curvature-sensing effects. Although there could be many other model variations are possible, our results suggest that curvature sensing is essential in constraining the effect of protein lateral diffusion on wave propagation, leading to sub-diffusive dependence of the wave speed on protein diffusion constant.

3.1 Alternative mechanochemical model with membrane tension-modulated F-BAR dynamics

This altered model scheme eliminates curvature-sensing effect. Instead, the F-BAR cortical recruitment is promoted only by the CWF module, and membrane tension speeds up the F-BAR turnover from the cortex. This way, Cdc42–N-WASP–F-BAR promotes actin polymerization, which increases the membrane tension that in turn triggers a faster F-BAR turnover from the cortex. This alternative scheme preserves the overall feedback

framework – the autocatalytic reactions of CWF intertwined with the actin-mediated negative feedback (Supplementary Fig. 11a). Additionally, this model is still mechanochemical in nature. Only the partial differential equation governing F-BAR dynamics is changed (see Eq. (3.1) below) and the timescale for actin dynamics is adjusted accordingly in order to obtain traveling waves (Eq. (3.2)). All other equations, approximations, and the model parameters were kept the same as in in the nominal case (Supplementary Note 1), except for the parameters listed in Supplementary Table 3. These parameters are also marked with * in the equations. We varied these parameters in order to obtain–traveling waves with wave speed and oscillation period that were reasonably consistent with the observed ones. These parameter variations may be beyond their respective physical ranges, as the altered model itself may not reflect the real physical mechanism of our traveling wave. The main purpose of this exercise – as well as those in the other alternative models (below) – was to explore the effect of eliminating curvature-sensing on the dependence of wave speed on protein lateral diffusions.

F-BAR dynamics:

$$\frac{\partial F}{\partial t} = \underbrace{D_F \cdot \nabla^2 F}_{\text{F-BAR diffusion}} + \left(\underbrace{k_{on}^{F*} \cdot (C+W)}_{\substack{\text{Cortical recruitment of F-BAR} \\ \text{by Cdc42 and N-WASP}}} \right) \cdot (1-F) - \underbrace{k_{off}^{F*} \cdot \frac{\sigma_M}{\sigma_0} \cdot F}_{\text{F-BAR turnover}} \tag{3.1}$$

Actin dynamics:

$$\frac{\partial A}{\partial t} = \lambda_A^* \cdot \left(\underbrace{\left(k_{on}^{A} \cdot \frac{(R/R_0)^4}{1+(R/R_0)^4} \right) \cdot (1-A)}_{\text{Arp2/3 mediated actin polymerization}} - \underbrace{k_{off}^{A} \cdot A}_{\text{Actin depolymerization}} \right) \tag{3.2}$$

3.2 Alternative mechanochemical model with membrane height-mediated F-BAR cortical recruitment

The altered model scheme, again, is without curvature-sensing effect. However, it preserves the overall feedback framework: The recruitment of cortical proteins feeds back to the membrane height *h*, rather than the Laplacian of the membrane height (Supplementary Fig. 11b). The membrane height variation can effectively change the cell volume and/or surface area, whose corresponding conjugates are osmotic pressure and/or membrane tension. These alternative schemes could therefore represent cases where osmotic pressure and/or membrane tension modulate cortical protein recruitment. In this sense, this altered model still involves mechanochemical feedback. Only the partial differential equation governing F-BAR dynamics is changed (see Eq. (3.3) below). All other equations, approximations, and model parameters were kept the same as in in the

nominal case in Supplementary Note 1, except for the parameters listed in Supplementary Table 3. These parameters are also marked with * in the equations.

F-BAR dynamics:

$$\frac{\partial F}{\partial t} = \underbrace{D_F \cdot \nabla^2 F}_{\text{F-BAR diffusion}} + \left(\underbrace{k_{on}^{F} \cdot (C+W)}_{\substack{\text{Cortical recruitment of F-BAR}\\ \text{by Cdc42 and N-WASP}}} + \underbrace{k_{on}^{Fh} \cdot \left(e^{\left(h/h_{00}^{*}\right)} - 1 \right)}_{\substack{\text{Membrane height induced}\\ \text{F-BAR cortical recruitment}}} \right) \cdot (1-F) - \underbrace{k_{off}^{F} \cdot F}_{\text{F-BAR turnover}}$$

(3.3)

3.3 Mechanochemical model with actin polymerization directly strengthening membrane-substrate adhesion

In this altered model scheme, actin polymerization directly increases the membrane-substrate adhesion energy, instead of membrane tension (Supplementary Fig. 11c). Here, the curvature-sensing effect is preserved. Only the partial differential equation governing membrane dynamics is changed (see Eq. (3.4) below), where h is the local membrane height, and the actin level increases the membrane-substrate adhesion (*i.e.*, the last term in the equation). All other equations, approximations and parameters were kept the same as in Supplementary Note 1.

Membrane dynamics:

$$\frac{\partial h}{\partial t} = \frac{1}{\lambda_m} \cdot \left[2\sigma_0 \cdot \nabla^2 h - 2\kappa_m \nabla^4 h + 2\kappa_m \Omega_F \nabla^2 F + f_F \cdot F - \tilde{k}_0 \cdot h \cdot (1 + \alpha_A \frac{(A/A_0)^3}{1+(A/A_0)^3}) \right]$$

(3.4)

3.4 Model with a simplified CWF module

In this model variation, the effects of the three components, Cdc42, N-WASP, and F-BAR were simplified as one lump-sum variable, Z (Supplementary Fig. 11d). Importantly, curvature-sensing effect is preserved. See Supplementary Note 2, Eq. (2.1)-(2.2) for details.

3.5 Model without term (1-X)

This model scheme is the same as our standard model presented except the terms *(1-X)* in the equations were changed to 1 (see Eqs. (3.5-3.9) below, and Supplementary Fig. 11e), where X stands for the variables *C, W, F, R, A* in the model. Importantly, curvature-sensing effect is preserved. The parameters were kept the same as in the nominal case except for those listed in Supplementary Table 3. These parameters are also marked with * in the equations.

Cdc42 dynamics:

$$\frac{\partial C}{\partial t} = \underbrace{D_C \cdot \nabla^2 C}_{\text{Cdc42 diffusion}} + \left(\underbrace{s}_{\text{Activation signal}} + \underbrace{k_{on}^{C} \cdot (W + F)}_{\text{Cortical recruitment of Cdc42 by N-WASP and F-BAR}} \right) - \underbrace{k_{off}^{C} \cdot C}_{\text{Cdc42 turnover}}$$

(3.5)

N-WASP dynamics:

$$\frac{\partial W}{\partial t} = \underbrace{D_W \cdot \nabla^2 W}_{\text{N-WASP diffusion}} + \underbrace{k_{on}^{W} \cdot (C + F)}_{\text{Cortical recruitment of N-WASP by Cdc42 and F-BAR}} - \underbrace{k_{off}^{W} \cdot W}_{\text{N-WASP turnover}}$$

(3.6)

F-BAR dynamics:

$$\frac{\partial F}{\partial t} = \underbrace{D_F \cdot \nabla^2 F}_{\text{F-BAR diffusion}} + \left(\underbrace{k_{on}^{F} \cdot (C + W)}_{\text{Cortical recruitment of F-BAR by Cdc42 and N-WASP}} + \underbrace{k_{on}^{Fh*} \cdot \left(e^{(\Omega_{h+}/\Omega_0)} - 1 \right)}_{\text{Membrane curvature induced F-BAR cortical recruitment}} \right) - \underbrace{k_{off}^{F} \cdot F}_{\text{F-BAR turnover}}$$

(3.7)

Arp2/3 dynamics:

$$\frac{\partial R}{\partial t} = \underbrace{D_R \cdot \nabla^2 R}_{\text{Arp2/3 diffusion}} + \underbrace{\left(k_{on}^{R} \cdot \frac{(W/W_0)^2}{1 + (W/W_0)^2} \right)}_{\text{N-WASP-dependent Arp2/3 activation}} - \underbrace{k_{off}^{R} \cdot R}_{\text{Arp2/3 inactivation}}$$

(3.8)

Actin dynamics:

$$\frac{\partial A}{\partial t} = \underbrace{\left(k_{on}^{A} \cdot \frac{(R/R_0)^4}{1 + (R/R_0)^4} \right)}_{\text{Arp2/3 mediated actin polymerization}} - \underbrace{k_{off}^{A} \cdot A}_{\text{Actin depolymerization}}$$

(3.9)

3.6 Model with less cooperative actin dynamics

In this altered model scheme, the Hill coefficient, n_A, in the equation for Arp2/3 complex-mediated F-actin polymerization was changed from 4 to 2 (see Eq. (3.10) below, and Supplementary Fig. 11f). All other equations, approximations and parameters were kept the same.

Actin dynamics:

$$\frac{\partial A}{\partial t} = \underbrace{\left(k_{on}^{A} \cdot \frac{\left(R / R_0\right)^{n_A}}{1+\left(R / R_0\right)^{n_A}} \right) \cdot \left(1-A\right)}_{\text{Arp2/3 mediated actin polymerization}} - \underbrace{k_{off}^{A} \cdot A}_{\text{Actin depolymerization}} \tag{3.10}$$

Supplementary References

1. Hui KL, Wang C, Grooman B, Wayt J, Upadhyaya A. Membrane Dynamics Correlate with Formation of Signaling Clusters during Cell Spreading. *Biophysical Journal* **102**, 1524-1533 (2012).

2. Simson R, Wallraff E, Faix J, Niewˆhner J, Gerisch Gn, Sackmann E. Membrane Bending Modulus and Adhesion Energy of Wild-Type and Mutant Cells of Dictyostelium Lacking Talin or Cortexillins. *Biophysical Journal* **74**, 514-522 (1998).

3. Bruinsma R, Behrisch A, Sackmann E. Adhesive switching of membranes: Experiment and theory. *Physical Review E* **61**, 4253-4267 (2000).

4. Dai J, Ting-Beall HP, Sheetz MP. The Secretion-coupled Endocytosis Correlates with Membrane Tension Changes in RBL 2H3 Cells. *The Journal of General Physiology* **110**, 1-10 (1997).

5. Lieber Arnon D, Yehudai-Resheff S, Barnhart Erin L, Theriot Julie A, Keren K. Membrane Tension in Rapidly Moving Cells Is Determined by Cytoskeletal Forces. *Current Biology* **23**, 1409-1417 (2013).

6. Krieg M*, et al.* Tensile forces govern germ-layer organization in zebrafish. *Nat Cell Biol* **10**, 429-436 (2008).

7. Bausch AR, Ziemann F, Boulbitch AA, Jacobson K, Sackmann E. Local Measurements of Viscoelastic Parameters of Adherent Cell Surfaces by Magnetic Bead Microrheometry. *Biophysical Journal* **75**, 2038-2049 (1998).

8. Bhatia VK*, et al.* Amphipathic motifs in BAR domains are essential for membrane curvature sensing. *EMBO J* **28**, 3303-3314 (2009).

9. Shimada A*, et al.* Curved EFC/F-BAR-Domain Dimers Are Joined End to End into a Filament for Membrane Invagination in Endocytosis. *Cell* **129**, 761-772 (2007).

10. Frost A*, et al.* Structural Basis of Membrane Invagination by F-BAR Domains. *Cell* **132**, 807-817 (2008).

11. Marco E, Wedlich-Soldner R, Li R, Altschuler SJ, Wu LF. Endocytosis Optimizes the Dynamic Localization of Membrane Proteins that Regulate Cortical Polarity. *Cell* **129**, 411-422 (2007).

12. Slaughter BD, Unruh JR, Das A, Smith SE, Rubinstein B, Li R. Non-uniform membrane diffusion enables steady-state cell polarization via vesicular trafficking. *Nat Commun* **4**, 1380 (2013).

13. Slaughter BD, Das A, Schwartz JW, Rubinstein B, Li R. Dual Modes of Cdc42 Recycling Fine-Tune Polarized Morphogenesis. *Developmental Cell* **17**, 823-835 (2009).

14. Das S, Yin T, Yang Q, Zhang J, Wu YI, Yu J. Single-molecule tracking of small GTPase Rac1 uncovers spatial regulation of membrane translocation and mechanism for polarized signaling. *Proceedings of the National Academy of Sciences* **112**, E267-E276 (2015).

15. Millius A, Watanabe N, Weiner OD. Diffusion, capture and recycling of SCAR/WAVE and Arp2/3 complexes observed in cells by single-molecule imaging. *Journal of Cell Science* **125**, 1165-1176 (2012).

16. Ramesh P, Baroji YF, Reihani SNS, Stamou D, Oddershede LB, Bendix PM. FBAR Syndapin 1 recognizes and stabilizes highly curved tubular membranes in a concentration dependent manner. *Sci Rep* **3**, (2013).

17. Zhu C, Das Sovan L, Baumgart T. Nonlinear Sorting, Curvature Generation, and Crowding of Endophilin N-BAR on Tubular Membranes. *Biophysical Journal* **102**, 1837-1845 (2012).

18. Freisinger T*, et al.* Establishment of a robust single axis of cell polarity by coupling multiple positive feedback loops. *Nat Commun* **4**, 1807 (2013).

19. Marchand JB, Kaiser DA, Pollard TD, Higgs HN. Interaction of WASP/Scar proteins with actin and vertebrate Arp2/3 complex. *Nature cell biology* **3**, 76-82 (2001).

20. Smith BA, Daugherty-Clarke K, Goode BL, Gelles J. Pathway of actin filament branch formation by Arp2/3 complex revealed by single-molecule imaging. *Proceedings of the National Academy of Sciences* **110**, 1285-1290 (2013).

21. Balcer HI, Daugherty-Clarke K, Goode BL. The p40/ARPC1 Subunit of Arp2/3 Complex Performs Multiple Essential Roles in WASp-regulated Actin Nucleation. *Journal of Biological Chemistry* **285**, 8481-8491 (2010).

22. Rohatgi R*, et al.* The Interaction between N-WASP and the Arp2/3 Complex Links Cdc42-Dependent Signals to Actin Assembly. *Cell* **97**, 221-231 (1999).

23. Bovellan M*, et al.* Cellular Control of Cortical Actin Nucleation. *Current Biology* **24**, 1628-1635 (2014).

24. Arasada R, Pollard Thomas D. Distinct Roles for F-BAR Proteins Cdc15p and Bzz1p in Actin Polymerization at Sites of Endocytosis in Fission Yeast. *Current Biology* **21**, 1450-1459 (2011).

25. Gerisch G*, et al.* Mobile actin clusters and traveling waves in cells recovering from actin depolymerization. *Biophysical Journal* **87**, 3493-3503 (2004).

26. Schoumacher M, Goldman RD, Louvard D, Vignjevic DM. Actin, microtubules, and vimentin intermediate filaments cooperate for elongation of invadopodia. *The Journal of Cell Biology* **189**, 541-556 (2010).

27. Rouiller I*, et al.* The structural basis of actin filament branching by the Arp2/3 complex. *The Journal of Cell Biology* **180**, 887-895 (2008).

28. Kunda P, Pelling AE, Liu T, Baum B. Moesin Controls Cortical Rigidity, Cell Rounding, and Spindle Morphogenesis during Mitosis. *Current Biology* **18**, 91-101 (2008).

29. Chaudhuri O, Parekh SH, Fletcher DA. Reversible stress softening of actin networks. *Nature* **445**, 295-298 (2007).

30. Charras GT, Hu C-K, Coughlin M, Mitchison TJ. Reassembly of contractile actin cortex in cell blebs. *The Journal of Cell Biology* **175**, 477-490 (2006).

31. Gilden J, Krummel MF. Control of cortical rigidity by the cytoskeleton: Emerging roles for septins. *Cytoskeleton* **67**, 477-486 (2010).

32. Hochmuth FM, Shao JY, Dai J, Sheetz MP. Deformation and flow of membrane into tethers extracted from neuronal growth cones. *Biophysical Journal* **70**, 358-369 (1996).

33. Harmandaris VA, Deserno M. A novel method for measuring the bending rigidity of model lipid membranes by simulating tethers. *The Journal of Chemical Physics* **125**, 204905 (2006).

34. Bermúdez H, Hammer DA, Discher DE. Effect of Bilayer Thickness on Membrane Bending Rigidity. *Langmuir* **20**, 540-543 (2004).

35. Takano K, Toyooka K, Suetsugu S. EFC/F-BAR proteins and the N-WASP-WIP complex induce membrane curvature-dependent actin polymerization. *EMBO J* **27**, 2817-2828 (2008).

36. Padrick SB, Doolittle LK, Brautigam CA, King DS, Rosen MK. Arp2/3 complex is bound and activated by two WASP proteins. *Proceedings of the National Academy of Sciences* **108**, E472-E479 (2011).

37. Pollard TD, Blanchoin L, Mullins RD. MOLECULAR MECHANISMS CONTROLLING ACTIN FILAMENT DYNAMICS IN NONMUSCLE CELLS. *Annual Review of Biophysics and Biomolecular Structure* **29**, 545-576 (2000).

38. Goley ED, Welch MD. The ARP2/3 complex: an actin nucleator comes of age. *Nat Rev Mol Cell Biol* **7**, 713-726 (2006).

39. Martinez-Quiles N*, et al.* WIP regulates N-WASP-mediated actin polymerization and filopodium formation. *Nat Cell Biol* **3**, 484-491 (2001).

40. Block J*, et al.* FMNL2 Drives Actin-Based Protrusion and Migration Downstream of Cdc42. *Current Biology* **22**, 1005-1012 (2012).

41. Schwartz MA, Luna EJ. How actin binds and assembles onto plasma membranes from Dictyostelium discoideum. *The Journal of Cell Biology* **107**, 201-209 (1988).

42. Fouchard J*, et al.* Three-dimensional cell body shape dictates the onset of traction force generation and growth of focal adhesions. *Proceedings of the National Academy of Sciences*, (2014).

43. Möhl C, Kirchgessner N, Schäfer C, Hoffmann B, Merkel R. Quantitative mapping of averaged focal adhesion dynamics in migrating cells by shape normalization. *Journal of Cell Science* **125**, 155-165 (2012).

44. Gallant ND, Michael KE, Garcia AJ. Cell Adhesion Strengthening: Contributions of Adhesive Area, Integrin Binding, and Focal Adhesion Assembly. *Mol Biol Cell* **16**, 4329-4340 (2005).

45. Jiang G, Giannone G, Critchley DR, Fukumoto E, Sheetz MP. Two-piconewton slip bond between fibronectin and the cytoskeleton depends on talin. *Nature* **424**, 334-337 (2003).

46. Wiseman PW*, et al.* Spatial mapping of integrin interactions and dynamics during cell migration by Image Correlation Microscopy. *Journal of Cell Science* **117**, 5521-5534 (2004).
