# Supplementary material for: Membrane shape-mediated wave propagation of cortical protein dynamics
Source: Nat Commun. 2018 Jan 10;9:136. doi: 10.1038/s41467-017-02469-1 (PMC5762918; doi:10.1038/s41467-017-02469-1)
Supplement: Supplementary file 3 — Description of Additional Supplementary Files [file 41467_2017_2469_MOESM3_ESM.pdf]

Description of Additional Supplementary Files

File Name: Supplementary Movie 1
Description: Zoom-in view of model results of the spatial-temporal patterns of F-BAR and membrane height in 1D cross-section corresponding to Fig. 2a.

File Name: Supplementary Movie 2
Description: Typical model result of the traveling wave. The mesh represents the membrane and heat map shows the level of F-BAR.

File Name: Supplementary Movie 3
Description: Waves of FBP17-EGFP and plasma membrane under TIRF and SRIC microscopy. Note the sequential formation of puncta in SRIC channel. The video is 20X faster than real time. The lookup table is inverted. Scale Bar: 20 μm.

File Name: Supplementary Movie 4
Description: Waves of FBP17-GFP stops at hypo-osmotic shock reversibly. Cells were perfused with normal buffer (300 mOsm) for 100 s and then changed hypo-osmotic (0.1X, 30 mOsm) buffer for 25 s twice. The video is 20X faster than real time. The lookup table is inverted. Scale Bar: 20 μm.

File Name: Supplementary Movie 5
Description: Waves of FBP17-GFP stops at hyper-osmotic shock reversibly. Cells were perfused with normal buffer (300 mOsm) for 100 s and then changed hyper-osmotic (2X, 600 mOsm) buffer for 25 s twice. The video is 20X faster than real time. The lookup table is inverted. Scale Bar: 20 μm.
